# Language and Emotion – A Foosball Study: The Influence of Affective State on Language Production in a Competitive Setting

**DOI:** 10.1371/journal.pone.0217419

**Published:** 2019-05-24

**Authors:** Nadine Braun, Martijn Goudbeek, Emiel Krahmer

**Affiliations:** Tilburg Center for Cognition and Communication, Department of Communication and Cognition, School of Humanities and Digital Sciences, Tilburg University, Tilburg, Netherlands; The University of Memphis, UNITED STATES

## Abstract

Our affective state is influenced by daily events and our interactions with other people, which, in turn, can affect the way we communicate. In two studies, we investigated the influence of experiencing success or failure in a foosball (table soccer) game on participants’ affective state and how this in turn influenced the way they report on the game itself. Winning or losing a match can further influence how they view their own team (compared to the opponent), which may also impact how they report on the match. In Study 1, we explored this by having participants play foosball matches in two dyads. They subsequently reported their affective state and team cohesiveness, and wrote two match reports, one from their own and one from their opponent’s perspective. Indeed, while the game generally improved participants’ moods, especially winning made them happier and more excited and losing made them more dejected, both in questionnaires and in the reports, which were analyzed with a word count tool. Study 2 experimentally investigated the effect of affective state on focus and distancing behavior. After the match, participants chose between preselected sentences (from Study 1) that differed in focus (mentioning the own vs. other team) or distancing (using *we* vs. the team name). Results show an effect for focus: winning participants preferred sentences that described their own performance positively while losing participants chose sentences that praised their opponent over negative sentences about themselves. No effect of distancing in pronoun use was found: winning and losing participants equally preferred the use of *we* vs. the use of their own team name. We discuss the implications of our findings with regard to models of language production, the self-serving bias, and the use of games to induce emotions in a natural way.

## 1 Introduction

Success and failure—everyone has experienced situations that ended one way or the other in their lives and knows how these situations made them feel. They influence our affective and mental state and can have an impact on our behavior and on how we communicate with others. In this paper, we investigated how success and failure in a competitive game alter participants’ affective states and how this subsequently influences how they describe the events that took place. In two studies, we used foosball (table soccer) as an immersive, naturalistic affect induction method and combined this with a task (e.g. writing a report) that was directly linked to the induction.

## 1.1 The influence of affect on language

The influence of our affective state on different aspects of language production has received some, but not much, scholarly attention. Some aspects of language production have already been shown to be influenced by a speaker’s/author’s affective state. By far the most thoroughly investigated is the relationship between affective state and changes in vocal expression. Among many others, Banse and Scherer [1], Owren and Bachorowski [2], and Goudbeek and Scherer [3] describe the acoustic cues that are associated with the speaker’s affective state and can be used to correctly identify those states.

Compared to the acoustic properties of affective speech, other aspects of language production, such as the linguistic form and content, have been much less studied. Notable exceptions are the work of Beukeboom and Semin [4], Forgas [5], and Pennebaker and colleagues (1993 [6] and further). For example, Beukeboom and Semin [4] found more abstract language in narratives that were written in a happy than in a sad mood, and Forgas explored the influence of affect on pragmatic aspects of behavior. Findings include, among others, that participants in a (mildly) negative mood formulated more polite requests than their happy counterparts [7] and that they conform more to Grice’s communication norms [8], such as quantity or relevance [9]. The work of Pennebaker and colleagues assesses, among other things, the effect of affective state on pronoun use. They found, for example, that depressed writers not only used more words with a lower valence than non-depressed people, but also used the pronoun “I” more than their non-depressed counterparts [10]. To automatically assess texts for different linguistic properties, including those related to affect, Pennebaker and Francis [11] developed the Linguistic Inquiry and Word Count (LIWC). LIWC provides word counts and percentages for words belonging to a multitude of different psychologically motivated word categories. Validated dictionaries exist for different languages, such as English [12], Dutch [13], and German [14] and have been periodically updated in recent years. LIWC covers a wide range of categories, such as positive and negative emotion words, negations, curse words, fillers, or words related to concepts like tentativeness or certainty.

What this handful of studies thus indicates is that affective state can influence aspects of language production beyond the acoustic level, although these studies are generally limited to specific linguistic categories (e.g., abstract words, personal pronouns). Overall, we still know very little about how affective states influence language production, partly because studying the relation between the two in an experimental setting is complicated.

### 1.2 Affect induction in a natural setting

To study the relationship between affect and language production, participants need to feel something in the first place. Often, affect induction happens through affect inducing stimuli such as pictures, music, or video clips, or by instructions to imagine or memorize an affectively laden event [15, 16]. While these methods have their particular strengths and weaknesses, they all have shown to be effective in eliciting affect, albeit to varying degrees. However, in almost all earlier studies, the relationship between the affect induction procedure and the subsequent task is tangential at best. Since the affective state as well as language are inherently intentional phenomena (e.g., they are about something; see, for example [17]), disconnecting the affect induction from its possible effects might obscure the relationship between the two.

Earlier work has shown that it is also possible to study the impact of affect on cognition outside of traditional laboratory. For example, Hastorf and Cantril [18] demonstrated that the perception of the fairness of an actual football game depended heavily on the affiliation of the viewers they asked to evaluate the game. Similarly, Cialdini and Borden [19] demonstrated how spectators of university football games included themselves in the successes of their teams by talking about won matches using “we” and wearing the respective university’s apparel more, which they both did to a much smaller extent after lost matches. Another strand of research, outside of the lab, concerned with the influence of affect on cognition (language in particular) is sentiment analysis. Sentiment analysis refers to the automatic categorization of texts into, for example, positive and negative, and has become a vast field of research to study attitudes towards brands, topics, or products online. To do this, researchers often use social network sites like Twitter to extract real users’ opinions and to identify trends (see [20, 21] as examples for sports). Although little is usually known about the authors in these studies, the cause for the users’ affective states and what they are writing about online are closely related.

To illustrate this connection between an author’s feelings and their language further, consider Krohne and Pieper [22]. They induced positive and negative moods through success and failure in a demanding cognitive task to investigate emotion regulation and coping disposition. After the induction, participants freely wrote down whatever came to their minds and, afterwards, their thoughts were coded for valence and relatedness. The authors found that, indeed, negative and positive thoughts that were related to the task were congruent with the induced moods: experiencing a failure led to overall more negative thoughts about the task, and experiencing a success led to more positive thoughts. Importantly, the overall number of thoughts and the number of positive, negative, or neutral thoughts that were completely unrelated to the task did not differ after successes or failures.

This study, while not primarily aimed at demonstrating such, shows that there is a close link between our affective state, its cause, and cognitions about the cause (see also [23–26]). This indicates the importance (when studying the influence of affective state on language production) of connecting the affect induction and the language production.

### 1.3 The influence of perspective on language

Studying the effect of positive and negative affect on language production and communication is further complicated by the potential mediating influence of perspective. Some studies show that negative affect worsens communication [27], while others show improvement [9, 28]. Crucially, the latter studies used video excerpts that were unrelated to the task to induce affect, while in the study of Baker-Ward and Eaton [27] affect induction happened in a natural social setting, and the task was closely related to the induction procedure. Baker-Ward and colleagues asked children who just won or lost a soccer match to describe the match they had played and found differences in the narratives, such as a more factual report for winning children compared to more interpretive descriptions for losing children. So, while the children ostensibly participated in the same match, what they talked about and especially *how* they talked about it differed. Since, following Bower [23] and Krohne and Pieper [22], our cognitions are congruent with our affective state, we can assume that the differences in the children’s narratives coincide with the changes in affective state because their perspectives on the soccer game, caused by winning or losing, and the narratives were closely related.

Hence, the study by Baker-Ward and Eaton [27] implies that our affective state is also inherently linked with the perspective we take in our narratives. The importance of perspective is additionally confirmed by the football study of Hastorf and Cantril [18]. As has been briefly addressed in 1.2, the study demonstrated how vastly different an event (in this case fairness of a football game) can be perceived depending on one’s point of view; answers to questions such as “Who initiated the rough play?” and “Was the game fair and clear?” depended greatly on the side the respondents were on (see also [29]). Likewise, perspective and affect do not only influence how an event–especially one ending in success or failure–is remembered and recounted, but also the role we assign to ourselves (and our team) in the outcome of the event.

### 1.4 Basking and distancing

The issues of perspective and affect that are inherent in success and failure also touch on the subject of causal attribution. In situations with positive or negative outcomes that can threaten one’s self-perception, implications for ourselves tend to be mediated by the self-serving bias. It postulates that people generally attribute successes more to their own performance and abilities, while distancing themselves from failures and attributing those to external causes [30–32]. This pattern occurs even when a person is not directly involved in the process leading up to the respective outcome. For example, Downs and Sundar [33] let participants play a virtual bowling match together with a team, which, they were told, consisted of random online game participants, against another online team. The outcome of the game was controlled in such a way that it ended in a win or a loss for the participant’s team. Importantly, only the top three (unbeknown to the participant, fictional) scores counted, which the participant’s score was never part of. This was even marked in the score board to ensure participants were aware of the fact that they did not have any influence on the outcome. Still, participants attributed successful games in parts to themselves, while blaming failures on their teammates.

Especially for large-scale events such as elections [34, 35] and sports events [19, 34, 36, 37], patterns such as “basking in reflected glory” (BIRGing, [19]) after successful outcomes and “cutting off reflected failure” (CORFing, [38]) after unsuccessful outcomes have been observed. Fans and supporters publicly proclaim their affiliation to a contestant or team more prominently after a success and withdraw more after a defeat. However, these tendencies are mediated by the strength of team affiliation [39]: in case of strong affiliation and long-time support, even negative events do not change fan and supporter behavior, whereas they do when affiliation is weak and support is relatively recent.

On a smaller scale, Snyder and Lassegard [38] studied distancing in a small group of students who were asked to present group work with their group after receiving positive or negative feedback. Even in a small group of peers, students preferred not to participate in the group presentation more often after negative feedback. This is supported by Wann and Hamlet [40], who demonstrated the moderating effect of affiliation as well as a distancing effect in smaller groups, also with regard to possible future failures. If distancing and basking are such common self-preservation and emotion regulation strategies in competition situations and if we assume that cognitive processes influence each other, it should be possible to trace both processes in descriptions of such an event.

These distancing strategies can also be linked to more recent work on emotion regulation and affect labelling [41]. While such studies usually move into the direction opposite of the one we are following in this paper by investigating the influence of language on emotions, we recognize some similarities. What Cialdini and Borden [33] identified as linguistic basking and distancing behavior relates to the phenomenon of psychological distancing, which, e.g., Nook and Schleider [42] have analyzed through language on three dimensions: physical (here vs. not here), temporal (now vs. not now), and social (I vs. not I), the latter pertaining to BIRGing and CORFing, respectively. They argued that linguistically distancing oneself from a negative stimulus serves as a form of emotion regulation and decreases experienced negative affect. This automatic engagement in psychological distancing, facilitated by language, as a means of emotion regulation has also been demonstrated in studies on (expressive) writing as a treatment for depression [43–45].

### 1.5 The current studies

Our goal is to study the influence of affective state on language production by introducing a new experimental paradigm in which the affect induction and language production are closely intertwined. In the current studies, we created a competitive game situation in which we have more control over the author and their relationship with the game than in professional sports reporting, social media posts, or the reporting on big events but which is more natural than traditional laboratory setups.

We use a foosball game to induce positive and negative affect related to winning and losing in a group: the participants are known, part of teams, and, importantly, they are the ones writing the reports. Due to the competitive nature of the game and people’s inherent dislike of failure, we expect the effects of winning and losing to be strong enough to influence the affective states of participants. This setup allows for affect to be induced in a more natural, social environment directly in the lab than in traditional studies, in which affect is induced with pictures, video clips, or emotional recall. With the connected writing task, we manage to collect a text corpus based on and related to the affect induction procedure, to investigate changes in written language and in the group dynamics as a function of affect. We expect to find differences in a variety of text properties of written foosball match reports based on positive and negative affect, operationalized as game outcome, specifically with regard to emotionality and perspective.

In the following, we present two studies: Study 1 explores different aspects of the language participants used in their written reports of the foosball games they played. In this study, our focus is on finding different communicative strategies depending on game outcome, which indicates affect. We will use LIWC categories and investigate linguistic references to the foosball teams involved in the form of pronouns and team names to explore the foosball reports written by participants to assess the effect on affective word use and linguistic focus (i.e., referring to one’s own team or the other team). In Study 2, we focus on two of these aspects in more detail, namely the effect of affect through winning or losing on the use of focus and linguistic distancing behavior (e.g., using “we” in the case of winning, but the team name when losing).

## 2 Study 1

### 2.1 Design

The experiment in the first study consisted of a naturalistic emotion induction procedure with two rounds of foosball games and a connected writing task. Variables of interest were self-reported affective state and distancing and basking, as well as 13 LIWC categories derived from the reports written about the matches. Reports were written from the own team’s and the other team’s perspectives. Affective state and basking/distancing were assessed using questionnaires, and basking/distancing was additionally annotated in the reports in the form of occurrences of the pronoun “we” and of team names. Since the questionnaires do not afford the manipulation of perspective, only outcome and time could effect changes. For the language in the reports, both the effects of outcome and perspective were assessed.

### 2.2 Participants

In total, 114 participants (74 women), age ranging from 18 to 40 years (*M* = 21.6), took part in the study. Participants were students and junior staff, mostly PhD students and young post-docs, of Tilburg University who participated for course credit or candy. All were fluent speakers of Dutch and partook in groups of four, with each team consisting of two people. We opted for same-sex groups in order to observe possible gender effects and to avoid within team interactions thereof. Two times, confederates were used due to no-shows of signed-up participants. All but 4 participants had played foosball before and were familiar with the game.

### 2.3 Measures

We used several measures to determine possible relationships between the outcome of the game, the participants’ affective states, and their writing style.

#### 2.3.1 Emotion questionnaire

In the competitive setting of a foosball game, many different emotions can appear. According to Holbrook and Chestnut [46], “an orientation toward victory or beating the opponent removes some games from the context of pure play or leisure by introducing an element of intrinsic motivation or work”. In order to account for the competitive aspect of the setup, we opted for a questionnaire aimed at athletes in sports events, the Sport Emotion Questionnaire [47]. The questionnaire includes 22 items that are combined into five affective categories. The original English questionnaire was translated to Dutch by a Dutch native speaker (different from the authors). The original categories in English are anger (irritated, furious, annoyed, angry; Cronbach’s α = .72 [Dutch translation]), anxiety (uneasy, tense, nervous, apprehensive, anxious; Cronbach’s α = .67 [Dutch translation]), dejection (upset, sad, unhappy, disappointed; Cronbach’s α = .70 [Dutch translation]), excitement (exhilarated, excited, enthusiastic, energetic; Cronbach’s α = .87 [Dutch translation]), and happiness (pleased, joyful, happy, cheerful; Cronbach’s α = .88 [Dutch translation]).

#### 2.3.2 Basking and distancing questionnaire

For self-report measures on distancing and basking behavior, we used a scale based on items created by Downs and Sundar [33]. All items were translated to Dutch by the same Dutch native speaker. Items for basking include: (1) I personally feel like I have won when our team wins. (2) I feel a sense of achievement because the team did well. (3) I have feelings of pride because the team did well. (4) I feel like my teammate played well. (5) I feel like we are winners. Items for distancing include: (1) I feel humiliated when the team loses (In the original study by Downs and Sundar [30], the item is phrased as “I feel humiliated because the team lost.” Since every participant saw all items regardless of the game outcome, this formulation would be illogical after a won match and was therefore changed to fit every outcome). (2) I do not feel any connection with my teammate. (3) I feel like my teammate played poorly. (4) I feel like my teammate is a loser. (5) I feel like my teammate did not play well. Additions to the scale were: (1) I feel like the outcome was a matter of luck. (2) I feel like I played well. (3) I enjoyed the game. These were added for different reasons. Distancing is not only possible by blaming bad performances on the teammates, but also by putting blame on outer circumstances, such as luck. Hence, item 1 was added to control for this. In a pilot of the study, participants indicated that they felt suspicious about being only asked about their teammate, but not about their own performance and experience, which ultimately led to the addition of item 2 and 3. To find the most reliable scale, first correlations of all items were calculated. The items with the highest correlations were then combined into a scale and controlled for reliability. For basking, a seven-item scale emerged including the five original items and item 2 and 3 from the additions with a good reliability (Cronbach’s α = 0.83 [Dutch translation]). For the distancing scale a 4-item measure emerged including items 2, 3, 4, and 5 with an acceptable reliability (Cronbach’s α = 0.73 [Dutch translation]). Item 1 was excluded from the scale because of low, non-significant correlations with the other distancing items. This seems reasonable, though: in order to feel humiliated by the team’s performance, there must be an emotional link to the team. If a participant does not feel connected to their team, or only weakly so, there is no reason for them to feel humiliated after a lost match because they will not feel as responsible or connected to the outcome either.

Overall, a correlation analysis confirmed that the basking and distancing scales were indeed sufficiently negatively correlated, *r* = -.52, *p* < .001, N = 226.

#### 2.3.3 Match reports

After each match, participants wrote two reports about the matches they just played, one from their own perspective and one from the perspective of the other team. They were requested to write at least about 50 words and they obtained an error message when they submitted less than 250 characters. Otherwise, there were no restrictions on length or style. They were given the following instruction (translated from Dutch; perspective originally marked in bold red):

*Imagine you had to write an online report for the match you just played for the fans of **your/the other team***. *Of course, they would like to experience what happened and how it was. What can you tell them about it? What happened in the game? How was your opponent? What can you tell them about **your/the other team’s** performance? Do you have any thoughts on the following match?*

To encourage them to write an extensive report, participants were informed that the best reports would enter a lottery for a gift voucher. The instructions were counterbalanced for perspective to avoid possible order effects. In each team, one person started with the report for their own team and one with the report for the other team per round. Time taken to write the reports was recorded. The reports were analyzed with LIWC, focusing on thirteen pre-determined affect-related categories [48], such as positive and negative emotion words, words signaling specific emotions (anger, sadness, anxiety), and words referring to achievements (e.g., “earn”, “hero”). Aside from the affect related categories, we will also consider pronouns, where first person plural is linked to in-groups and social connections while third person plural hints at out-group awareness; negations, which are linked to inhibition and uncertainty; certainty (e.g., “always”, “never”), tentativeness (e.g., “maybe”, “perhaps”, “guess”), inhibition (e.g., “constrain”, “stop”, “block”), and discrepancy (e.g., “should”, “would”, “could”), which are connected to emotional stability; and exclamation marks, which have been shown to be an indicator of positive affect in text-based communication [49].

In addition, the reports were manually coded by a Dutch native speaker, different from the authors, for references to the own and other team to investigate the main focus of the reports. Manual annotation was chosen over automated annotation to clearly distinguish referents, e.g., “they” could refer to either the own team or other team and team names could be shortened to nicknames or mistyped. The coding scheme was simple: every reference to the own team was marked as “own” and every reference to the other team as “other”. These references could take different forms, such as pronouns (separated into we/our/ours and they/their/them) or lexical references (team names or nicknames). First person plural “own” references were regarded as basking and other references to the own team were considered more distant options. Determining which reference referred to which team was straightforward.

#### 2.3.4 Demographics

Participants were asked to indicate their gender, age, whether they were native speakers of Dutch, and any other languages they spoke. In addition, they had to indicate whether they had a language disorder like dyslexia. Further, they were asked whether they knew their own teammate or one or both of the participants in the other team. Finally, they specified their experience with foosball, so whether they had played the game before and how frequently they played on a scale from 1 to 5 (Very rarely–Very often). Nearly all participants indicated that they had played foosball only occasionally, hence we do not analyze this any further.

### 2.4 Material and procedure

Before the beginning of a session, the room was prepared and the camera set up. The survey for the experiment was preloaded in the browsers of all four computers and the participant numbers entered by the experimenter.

**Fig 1:**
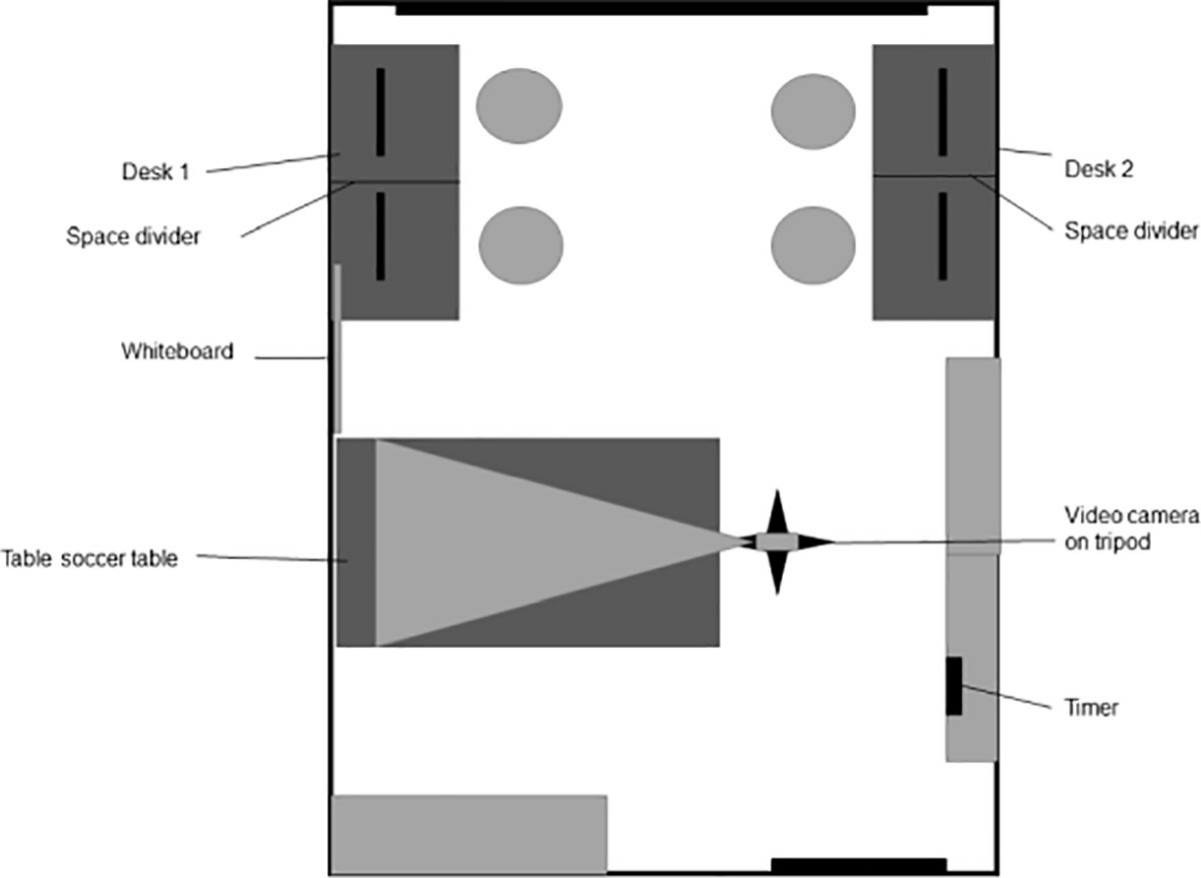
Overview of the experiment room (bird’s eye view).

**Fig 2:**
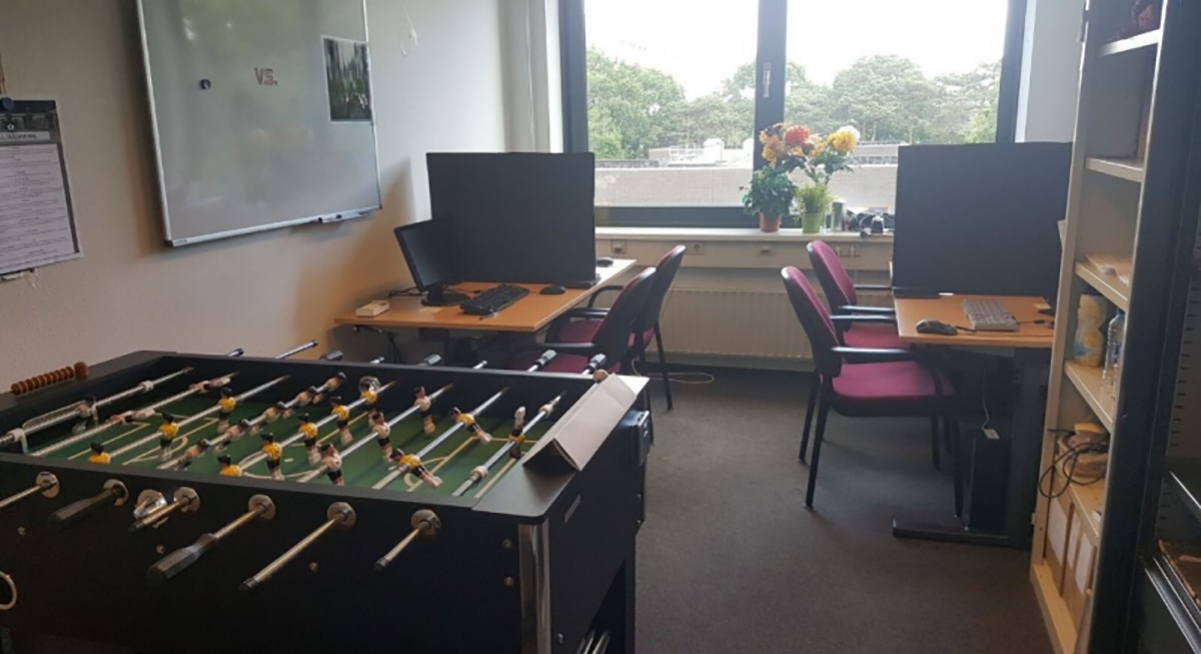
Actual experiment room.

As soon as all four participants had arrived, they were asked to draw one of four cards that were offered to them by the experimenter before entering the room. The cards, which had the letters A, B, C, and D written on them, determined which participants were going to form a team. This procedure was chosen to assign groups as randomly as possible due to a likely variation in skill levels. The participants then entered the room with the experimenter and the teammates were placed next to each other at work stations at two desks, which had the same letters as the cards attached to them to signal each participant where to sit. The desks with the teams faced away from each other and the computers of the individual participants were separated with a screen (see Figs 1 and 2).

The experimenter briefly explained the procedure, and, if no questions remained, the participants were asked to read and sign the informed consent forms. Afterwards, the participants were asked to come up with a name for their team, which was then written on a whiteboard (“Team A vs. Team B”). This step was included to induce the affiliation between team members and to stress the social aspect of the game.

Next, a coin toss determined the sides and colors at the foosball table and who would start the first game. If no questions arose, participants filled in the first part of the survey, which included questions about the team names, the team colors, and the Sport Emotion Questionnaire. The experimenter stayed in the room in case technical problems with the survey arose.

Upon completing the first part, participants proceeded to the foosball table. They were told that their position in the first game, attack or defense, did not matter because they would switch in the second round. A camera was set up to record the voices of the participants and the table surface during the game. As a guideline, the official rules of foosball were printed on a poster next to the table. The timer was set to 8 minutes and the experimenter left the room.

When the timer signaled the end of the round, the experimenter re-entered the room, turned off timer and camera, and checked the outcome on the whiteboard. If the game was tied, participants were told to play one match ball with the experimenter present to determine a winner. If there was a winner, the team was congratulated on the victory and could choose their prize from a box with a variety of candy. The teams were then asked to take a seat again and fill in the second part of the survey. After giving information about the outcome, they were first asked to write the two match reports before completing the Sport Emotion Questionnaire and the Basking and Distancing Questionnaire. In this regard, having participants complete the emotion questionnaire after the writing task was completed was a conscious choice: in order to yield the most impact of post-game emotions on the match reports, we decided not make participants aware of their emotions and not to prime them with specific emotion terms before the writing task.

After everyone completed the second part of the survey, the teams returned to the foosball table and played the second match. The procedure was the same as in round one, except that participants switched attack and defense at the table within the teams. Two rounds were chosen to allow participants to take both positions at the table, hence eliminating the position being used to explain bad performances, and to have a complete foosball “season” (home & away), similar to soccer and other sports seasons.

After the last round, when the winning team had been awarded their prize, the teams returned to the desks to finish the survey. The procedure was the same as in the first round, except for some additional demographic items in the end.

When everyone had finished the survey, participants received the debriefing form. If one team lost both rounds of table soccer, they were also offered candy. The experimenter then thanked the participants for their participation and finished the session.

This study was carried out in accordance with the recommendations of the Tilburg School of Humanities and Digital Sciences Research Ethics and Data Management Committee with written informed consent from all subjects. All subjects gave written informed consent in accordance with the Declaration of Helsinki. The protocol was approved by the Tilburg School of Humanities and Digital Sciences Research Ethics and Data Management Committee.

### 2.5 Results

#### 2.5.1 Analysis strategy

The individual 22 items of the emotion questionnaire were collapsed into the 5 emotions categories (anger, anxiety, dejection, excitement, and happiness) and the 11 items of the basking and distancing questionnaire were combined into “basking” and “distancing” measures.

Since game outcome could change for each participant (e.g., losing the first round but winning the second and vice versa) during an experiment session, analyzing the self-reported data with a simple analysis of variance was not possible. Hence, we analyzed the self-reported emotions and the basking and distancing questionnaire with linear mixed effects modelling in R using the LMER function of the lme4 package [50] to account for the multilevel nature of the data.

Following Winter [51], we started with a bottom-up approach for building the models with outcome as the predictor and participants as random intercepts. The models for all emotions and basking/distancing converged. In a next step, we introduced a full random effects structure by adding participants as random slopes. However, these models did not converge, most likely due to the number of observations being too small. In addition, we controlled whether each model improved by adding time, gender, age, and familiarity with other participants and the own teammate. To determine the improvement of model fit, the full models were compared to reduced models, which did not contain the respective fixed effect, in an ANOVA. For example:

Dejection∼Outcome+Gender+(1|Participant)

This full model on Dejection was compared to the following reduced model:

Dejection∼Outcome+(1|Participant)

After building the models, we used parametric bootstrapping over 100 iterations to estimate confidence intervals, standard errors, and *p*-values on the final models.

In addition to the self-reported measures, we give an overview of the descriptive statistics and the exploratory LIWC analyses for thirteen LIWC categories (Positive emotion, Negative emotion, Sadness, Anxiety, Anger, Negation, Achievement, Pronoun, Certainty, Inhibition, Tentativeness, Discrepancy, Exclamation marks). However, as we did not formulate hypotheses about the spread of all LIWC categories, we will not provide significance values and refrain from performing inferential statistical analyses.

#### 2.5.2 Affective variables

**Sport emotion questionnaire.** For an overview of means and standard deviations of all emotions, see Table 1. Compared to won matches (dejection: *M* = 1.06, *SD* = 0.21; anger: *M* = 1.06, *SD* = 0.22; excitement: *M* = 3.67, *SD* = 0.91; happiness: *M* = 3.89, *SD* = 0.74; anxiety: *M* = 1.15, *SD* = 0.27), self-reported emotions after lost matches differed significantly for dejection (*M* = 1.28, *SD* = 0.40, *b* = 0.25, *SE* = 0.03, BC 95% CI [0.18, 0.32]), anger (*M* = 1.17, *SD* = 0.36, *b* = 0.11, *SE* = 0.03, BC 95% CI [0.05, 0.19]), excitement (*M* = 3.13, *SD* = 0.97, *b* = -0.39, *SE* = 0.08, BC 95% CI [-0.55, -0.22]), and happiness (*M* = 3.27, *SD* = 0.96, *b* = -0.48, *SE* = 0.08, BC 95% CI [-0.65, -0.32]), indicating that winning made participants happier and more exited, while they felt more dejected and angrier after losing. Adding familiarity with the teammate or the other participants in the group or demographics (age, gender) did not improve the models.

**Table 1:** Descriptive statistics for emotions and basking/distancing in baseline, loss, and win.

	BASELINE	WIN	LOSS
	M (SD)	M (SD)	M (SD)
Anger	1.11 (0.28)	1.06 (0.22)	1.17 (0.36)
Anxiety	1.36 (0.44)	1.15 (0.27)	1.17 (0.37)
Dejection	1.14 (0.36)	1.06 (0.21)	1.28 (0.40)
Excitement	3.05 (0.85)	3.67 (0.91)	3.13 (0.97)
Happiness	3.53 (0.83)	3.89 (0.74)	3.27 (0.96)
Basking	—	4.44 (0.44)	3.43 (0.75)
Distancing	—	1.50 (0.59)	1.93 (0.81)

Anxiety (Baseline: *M* = 1.36, *SD* = 0.44; Round 1: *M* = 1.22, *SD* = 0.38; Round 2: *M* = 1.10, *SD* = 0.23) was only predicted by time (Intercept: *b* = 1.36, *SE* = 0.03, BC 95% CI [1.29, 1.43]; Round1: *b* = -0.14, *SE* = 0.03, BC 95% CI [-0.20, -0.078]; Round 2: *b* = -2.26, *SE* = 0.03, BC 95% CI [-0.32, -0.20]), but not by game outcome, suggesting that participants became gradually less anxious over the course of the experiment independent of the outcome of the matches. This was not the case for the other emotions, which were unaffected by time. For an overview of all models, see Table 2.

**Table 2:** Analyses for all five emotions (anxiety, dejection, excitement, happiness, anger).

	*B*	*SE b*	*95% CI*
	***Anxiety***		
Intercept	1.12	0.03	1.06, 1.20
Outcome Loss	0.06	0.04	-0.01, 0.16
	***Dejection***		
Intercept	1.04	0.03	0.98, 1.12
Outcome Loss	0.25	0.03	0.18, 0.32
	**Excitement**		
Intercept	3.59	0.09	3.40, 3.78
Outcome Loss	-0.39	0.08	-0.55, -0.22
	**Happiness**		
Intercept	3.82	0.08	3.65, 3.98
Outcome Loss	-0.48	0.08	-0.65, -0.32
	**Anger**		
Intercept	1.05	0.02	1.00, 1.11
Outcome Loss	0.11	0.03	0.05, 0.19

Estimated coefficients *B*, standard errors *SE b*, and 95% confidence intervals for the full mixed models; participants as random intercepts; game outcome as the predictor; win as the reference category.

**Basking and distancing questionnaire.** Self-reported basking as well as distancing significantly depends on game outcome (Table 3). While participants generally bask more than they distance themselves, reports of basking additionally increase after a won match (*M* = 4.44, *SD* = 0.44) compared to after a loss (*M* = 3.43, *SD* = 0.75, *b* = -0.99, *SE* = 0.08, BC 95% CI [-1.17, -0.84]). The opposite is true for distancing: participants distance themselves less after won matches (*M* = 1.50, *SD* = 0.59) than after lost matches (*M* = 1.93, *SD* = 0.81, *b* = 0.47, *SE* = 0.09, BC 95% CI [0.31, 0.67]). Additionally, adding familiarity with the teammate as a predictor improved the model for distancing: if participants knew their teammate, they distanced themselves less than when they did not know each other, which was not the case for basking. However, teammate familiarity did not interact with game outcome and, hence, did not change the effect. Adding familiarity with other participants or the demographics (gender, age) did not improve either model.

**Table 3:** Analyses for basking and distancing.

	*B*	*SE b*	*95% CI*
	**Basking**		
Intercept	4.43	0.06	4.32, 4.56
Outcome Loss	-0.99	0.08	-1.17, -0.84
	**Distancing**		
Intercept	1.47	0.07	1.31, 1.62
Outcome Loss	0.47	0.09	0.31, 0.67

Estimated coefficients B, standard errors SE b, and 95% confidence intervals for the full mixed models; participants as random intercepts; game outcome as the predictor.

As can be seen in Table 4, self-reported emotions and basking and distancing correlate as one would have expected: basking significantly correlates positively with excitement and happiness, while it correlates negatively with dejection and anger. Distancing, on the other hand, correlates positively with dejection and anger, and negatively with excitement and happiness.

**Table 4:** Correlations of self-reported emotions and self-reported basking and distancing.

		Anxiety	Dejection	Excitement	Anger	Happiness	Basking	Distancing
Anxiety	Pearson Correlation	1	.438**	-.008	.394**	-.043	-.029	.019
	Sig. (2-tailed)		.000	.872	.000	.367	.533	.695
Dejection	Pearson Correlation	.438**	1	-.270**	.671**	-.359**	-.412**	.344**
	Sig. (2-tailed)	.000		.000	.000	.000	.000	.000
Excitement	Pearson Correlation	-.008	-.270**	1	-.226**	.806**	.522**	-.332**
	Sig. (2-tailed)	.872	.000		.000	.000	.000	.000
Anger	Pearson Correlation	.394**	.671**	-.226**	1	-.296**	-.350**	.366**
	Sig. (2-tailed)	.000	.000	.000		.000	.000	.000
Happiness	Pearson Correlation	-.043	-.359**	.806**	-.296**	1	.577**	-.388**
	Sig. (2-tailed)	.367	.000	.000	.000		.000	.000
Basking	Pearson Correlation	-.029	-.412**	.522**	-.350**	.577**	1	-.521**
	Sig. (2-tailed)	.533	.000	.000	.000	.000		.000
Distancing	Pearson Correlation	.019	.344**	-.332**	.366**	-.388**	-.521**	1
	Sig. (2-tailed)	.695	.000	.000	.000	.000	.000	

**. Correlation is significant at the 0.01 level (2-tailed).

N = 450 for all correlations.

#### 2.5.3 Match reports

**Basic descriptive statistics.** Overall, a total of 450 match reports consisting of 42,279 tokens (win: 21,465; loss: 20,814), with an average text length of 94.0 tokens/text, were written by the participants in an average of 194.8 seconds/text (Table 5). The shortest text is 33 tokens written in about 1.5 minutes, the longest 301 tokens written in almost 15 minutes. The vast majority of participants followed the requested 50 words-per-text limit, with only 26 reports ranging between 33–49 words. In general, male participants took almost one minute longer to write the texts, which were also about 10 words longer on average.

**Table 5:** Descriptive statistics of the match reports, classified into round (1/2), outcome (Win/Loss), and Perspective (own/other).

	Number of texts	Mean Text Length (SD)	Mean Time (SD)
**Round 1**	**226**	**97.2 (43.8)**	**223.3 (134.1)**
**LOSS**	**114**	**98.8 (43.9)**	**225.6 (142.7)**
Other	55	94.7 (39.3)	230.4 (138.2)
Own	59	102.6 (47.8)	221.2 (147.8)
**WIN**	**112**	**95.6 (43.8)**	**220.9 (125.3)**
Other	55	96.3 (48.7)	231.9 (142.9)
Own	57	94.8 (38.9)	210.2 (105.9)
**Round 2**	**224**	**90.7 (31.4)**	**166.6 (74.4)**
**LOSS**	**110**	**86.8 (30.1)**	**154.5 (67.6)**
Other	54	84.0 (28.7)	153.9 (76.2)
Own	56	89.6 (31.4)	155.0 (58.9)
**WIN**	**114**	**94.4 (32.3)**	**178.4 (78.9)**
Other	56	91.0 (27.8)	170.9 (67.7)
Own	58	97.7 (36.0)	185.6 (88.4)
**TOTAL**	450	94.0 (38.23)	194.8 (111.8)

Overall, text lengths and writing time did not vary much for reports about won and lost matches. With regard to game outcome, the reports written from the authors’ own perspective in the first round were similar in terms of number of words or writing time (Table 5). In the second round, however, writing time dropped, especially for reports about lost matches. Text length decreased for authors of losing teams assuming the winner’s perspective in the second round as well.

**Fig 3:**
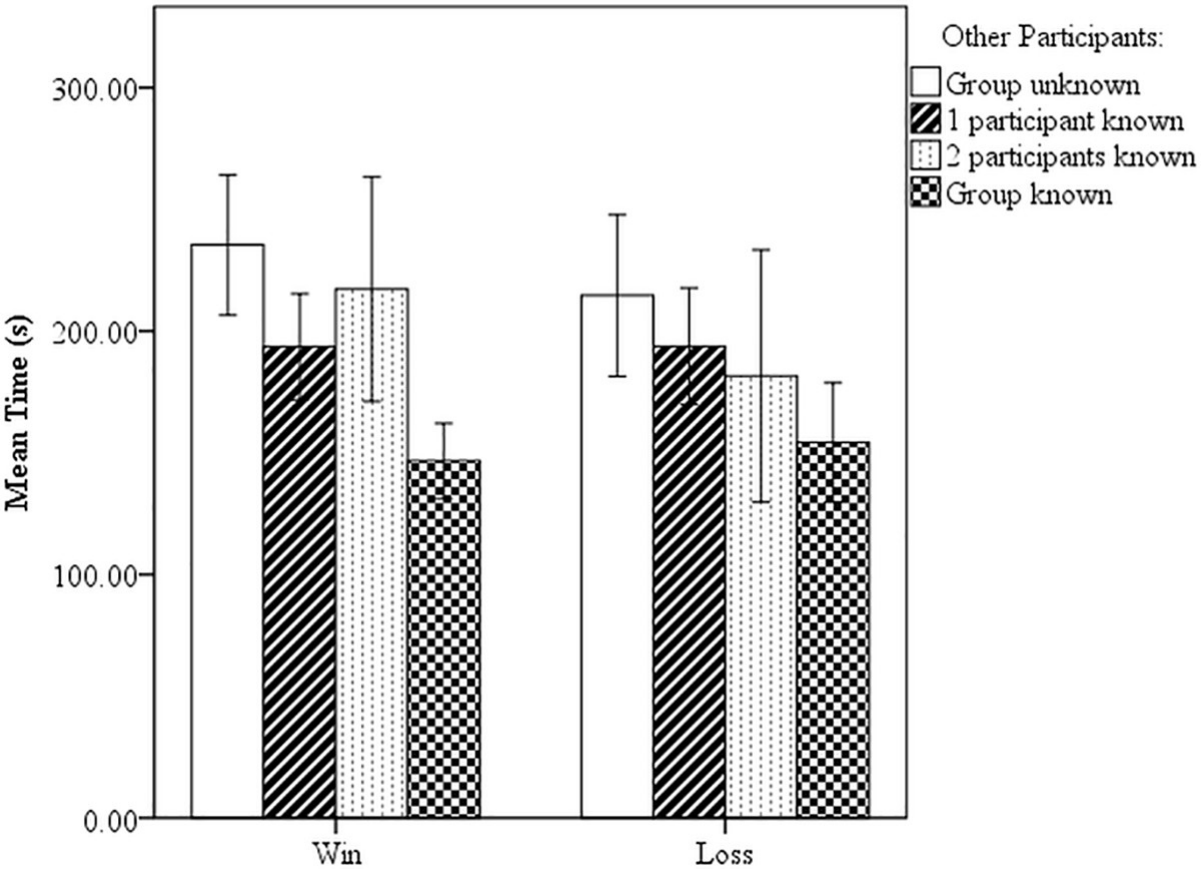
Average writing time related to familiarity of participants (group unknown–known to participant) for won and lost matches; error bars are 95% CIs.

**Fig 4:**
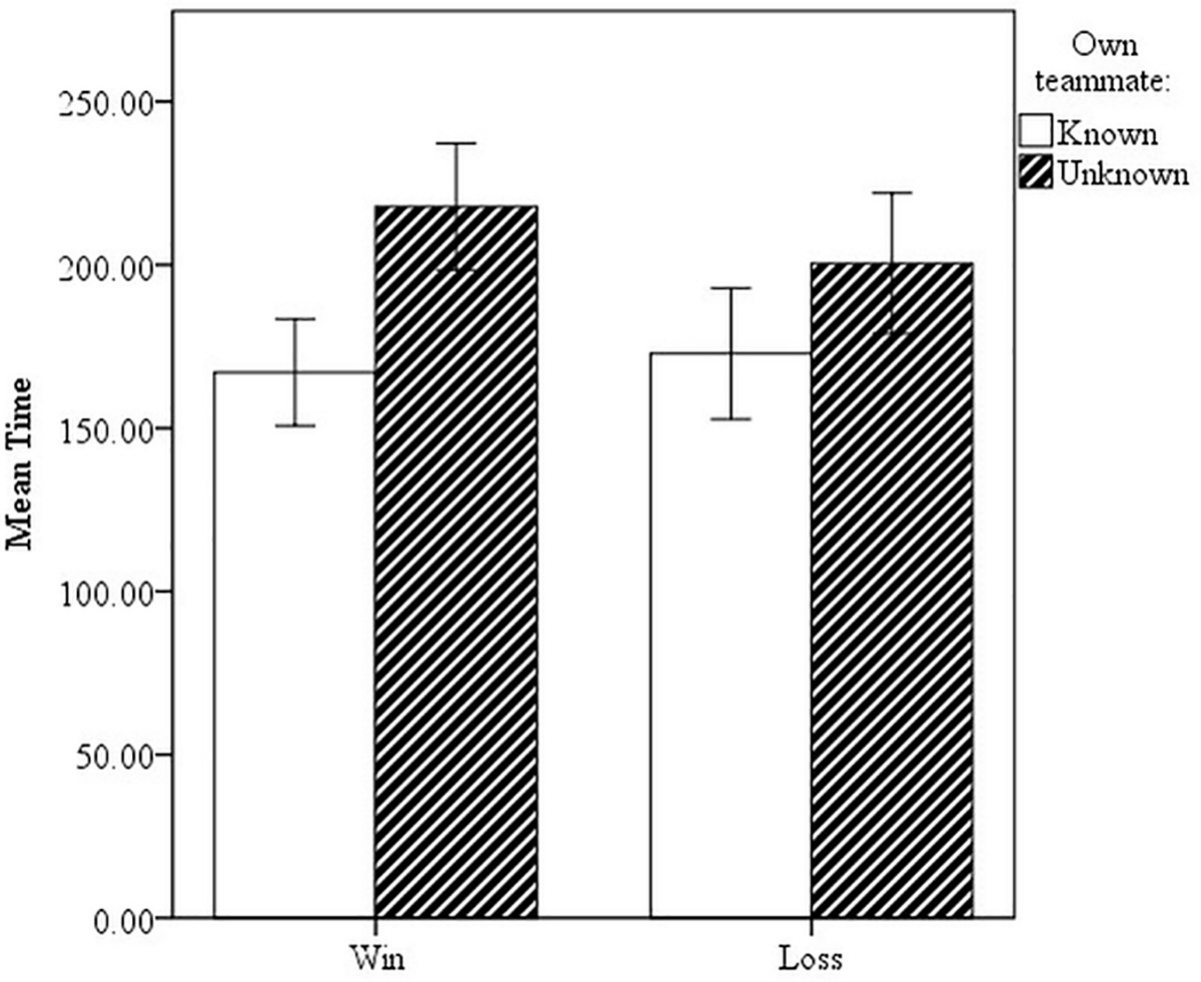
Average writing time related to familiarity of teammates (known/unknown) for won and lost matches; error bars are 95% CIs.

In addition, familiarity with the other participants affected the time taken to write the reports (Fig 3), while the average text length remained mostly unchanged. Writing time dropped (up to almost 1.5 minutes less) the more familiar the author was with the group (Win, group unknown: 235.4 s/ Win, whole group known: 146.6 s; Loss, group unknown: 214.6 s/ Loss, whole group known: 154.2 s). This pattern appeared independent of round and outcome. The same pattern emerges if we just look at familiarity with the own teammate: if teammates know each other, they take less time to write the reports in both rounds and both outcomes (Fig 4).

**LIWC.** Table 6 shows means and standard deviations for all LIWC categories mentioned in the introduction and Analysis Strategy sections.

**Table 6:** Means and standard deviations for LIWC categories in win and loss and perspective (own/other).

	WIN		LOSS	
	Own	Other	Own	Other
Positive emotion	3.39 (2.25)	2.92 (1.76)	3.13 (2.09)	3.97 (2.65)
Negative emotion	1.83 (1.56)	3.33 (2.21)	3.15 (2.16)	1.98 (1.64)
Sadness	0.18 (0.44)	0.79 (0.89)	0.90 (1.23)	0.24 (0.51)
Anxiety	0.12 (0.39)	0.15 (0.44)	0.12 (0.36)	0.14 (0.47)
Anger	1.04 (1.27)	0.98 (1.30)	0.97 (1.39)	1.05 (1.32)
Negation	0.98 (1.08)	0.79 (0.89)	1.49 (0.16)	1.03 (1.14)
Achievement	1.42 (1.45)	1.48 (1.14)	1.53 (1.61)	1.72 (1.60)
Pronoun	3.11 (2.42)	3.01 (2.37)	2.89 (2.38)	3.59 (3.23)
Certainty	0.79 (0.99)	0.79 (1.01)	0.74 (0.85)	0.86 (0.93)
Inhibition	0.37 (0.78)	0.34 (0.77)	0.29 (0.78)	0.37 (0.68)
Tentativeness	1.03 (1.09)	1.20 (1.16)	1.59 (1.39)	1.45 (1.58)
Discrepancy	1.64 (1.18)	2.26 (1.69)	2.16 (1.68)	2.01 (1.42)
Exclamation marks	0.78 (1.31)	0.35 (0.75)	0.39 (0.75)	0.69 (1.17)

As can be expected, there are more positive emotion words in reports about successful matches than in lost matches; even more so in reports by authors of the losing teams assuming the winners’ perspectives (Table 6). Sentences (1)–(3) are examples of expressions of positive affect in won matches:

(1) YES! We hebben weer een wedstrijd gewonnen jongens! (20C_R2_2T)

‘YES! We won another competition, guys!’

(2) De tegenstander had geen schijn van kans, en daar maakten wij mooi gebruik van. (10D_R1_2T)

‘The opponent did not stand a chance and we made good use of that.’

(3) Gelukkig herpakte OWN_TEAM zich na de 5e goal en mede dankzij goed teamwork en wat knap doelwerk kon OWN_TEAM uitlopen op team OTHER_TEAM. (22D_R1_2T)

‘Fortunately, OWN_TEAM got their act together after the 5^th^ goal and thanks to the good team work and nice goal work, OWN_TEAM could catch up with OTHER_TEAM.’

The opposite pattern occurs for negative emotion words: the proportion is lowest in reports about won matches by winning authors and highest when the winning authors assume the losers’ perspective. (4) to (7) illustrate negative affect after lost matches:

(4) Twee keer achter elkaar een vernedering door OTHER_TEAM, dat is in de voetbalgeschiedenis ongekend. (4B_R2_1T)

‘Humiliated two times in a row by OTHER_TEAM, that’s unheard of in the history of football.’

(5) Helaas weer een nederlaag… (27A_R2_2T)

‘Unfortunately, another defeat…’

(6) Wij nemen de schuld van deze nederlaag volledig op ons, het had namelijk veel beter kunnen gaan. (27A_R2_2T)

‘Only we are to blame for this defeat, it could have gone way better.’

(7) OWN_TEAM zal tijd nodig hebben deze ongelooflijke afgang te kunnen verwerken. (4B_R2_1T)

‘OWN_TEAM will need time to process this unbelievable flop.’

**Fig 5:**
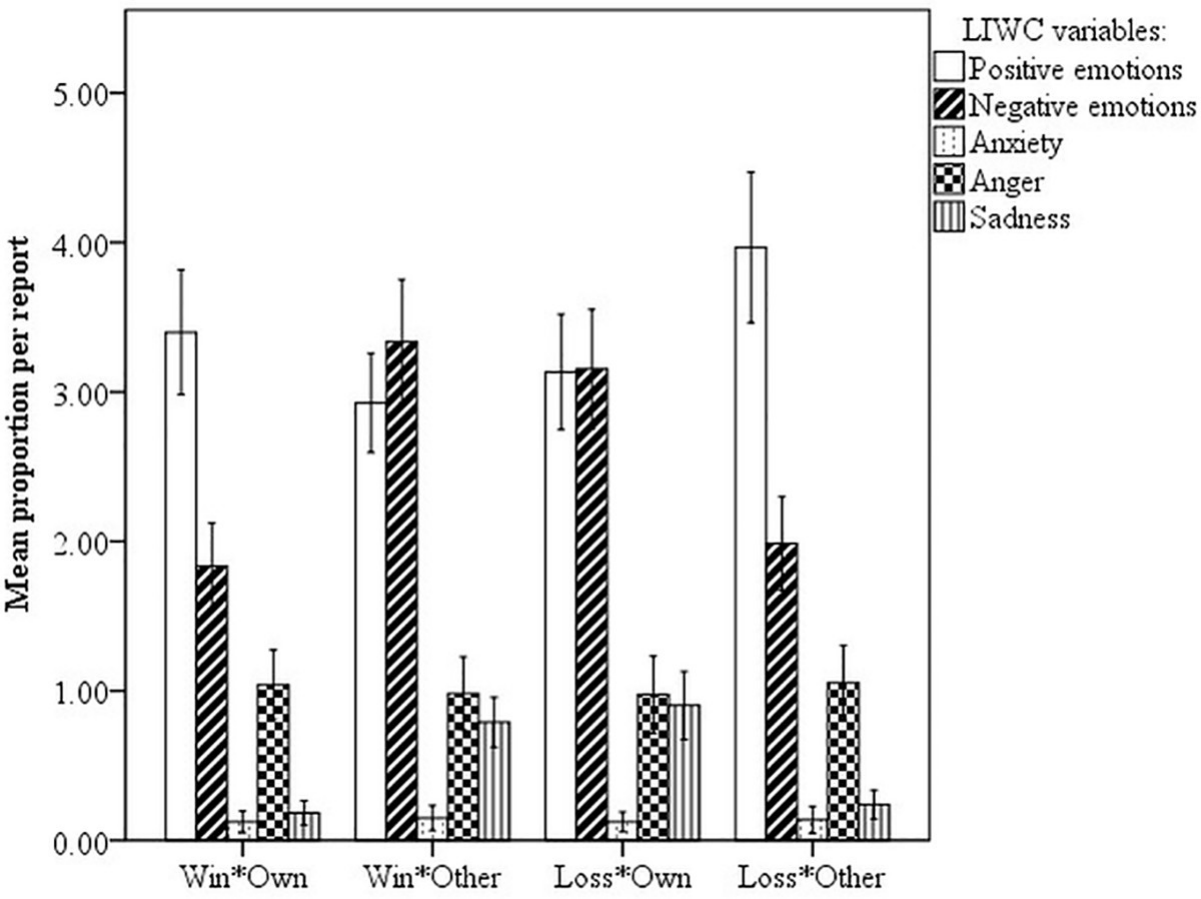
Mean proportions of LIWC emotion categories (positive emotion, negative emotion, anxiety, anger, and sadness) per match report by game outcome and perspective; error bars are 95% CIs.

Similarly, the proportion of words related to sadness is higher in reports on lost matches, both from the own and other perspective, than in reports on won matches. No apparent difference was found for words related to anxiety and anger. Overall, the affect occurring in the texts is similar to the results of the self-reported emotions (Fig 5).

However, in Fig 5, the proportion of positive emotion words is still high and almost balanced with negative emotion words in reports own matches of losing authors. By taking a closer look at the reports and LIWC’s annotations, we can identify two possible reasons for this: a) Some authors write positively about negative game outcomes, see (8) to (10), often focusing specifically on general game descriptions.

(8) Toch worden we hier niet door ontmoedigd en zijn we van mening dat de komende wedstrijden, met veel trainen, een stuk beter zullen gaan! (27A_R2_2T)

‘But we will not be discouraged by this and think that the following matches will be, with a lot of training, a lot better!’

(9) De kans is namelijk sterk aanwezig dat zij er de volgende keer met de winst vandoor gaan. Er is namelijk veel progressie te merken. (10B_R2_1T)

‘There is in fact a big chance that they will win next time. You can in fact see a lot of progress.’

(10) Verder verliep de wedstrijd goed, was het gezellig en wordt de volgende wedstrijd weer net zo spannend waarschijnlijk! (3A_R2_2T)

‘The match continued nicely, it was pleasant and the following match will probably be just as exciting.’

b) Due to the fact that LIWC annotates individual words, some negative implications are lost in the context of the text, see examples (11) to (12). For example, LIWC annotates the word *strong* as a positive emotion word in (11), although it is negative in the context of the sentence and the phrase *too strong*.

(11) Vooral de aanval van OTHER_TEAM was te sterk voor onze verdediging. (5A_R2_2T)

‘Especially the attack of OTHER_TEAM was too strong for our defense.’

(12) De defensie was bij de tegenstanders wel sterk. (3D_R2_1T)

‘The opponent’s defense was strong.’

(13) Het andere team hadden meer energie en misschien meer ervaring. (17D_R1_2T)

‘The other team had more energy and maybe more experience.’

On a similar note, irony and generally humorous descriptions of negative outcomes are almost impossible to pick up automatically with tools like LIWC, see (14).

(14) Zonde dat de verdediging het parerend vermogen had van een keukenpapiertje, waardoor het doelsaldo van de tegenstander drie keer dat aantal binnen wist te ketsen. (28C_R2_2OT)

‘It’s a shame that the defense had the paring capability of kitchen paper, which tripled the goal balance of the opponent.”

Losing participants generally used more negations, particularly in reports from their own perspective. This stylistic difference seems to be related to the affect and perspective induced by losing the match, since winning participants writing from the losers’ perspectives seemed not to be aware of this and did not adapt their writing accordingly.

Words related to achievements and pronouns were most used by losing authors assuming the winners’ perspectives (Table 6). In terms of pronouns, the proportion increased by almost one percent point from authors writing about their own lost matches to them pretending to be in the winning team. No big difference in the text categories was found for words relation to certainty and inhibition. The proportion of exclamation marks doubled in reports about won watches, either from actual winning authors or losing authors switching the perspective.

So, both perspectives adapted the use of exclamation marks according to game outcome. Also, words relating to tentativeness were generally higher in losing authors’ reports. Winning authors used more words relating to discrepancy when assuming the other team’s perspective, even more so than the losing authors themselves.

**Fig 6:**
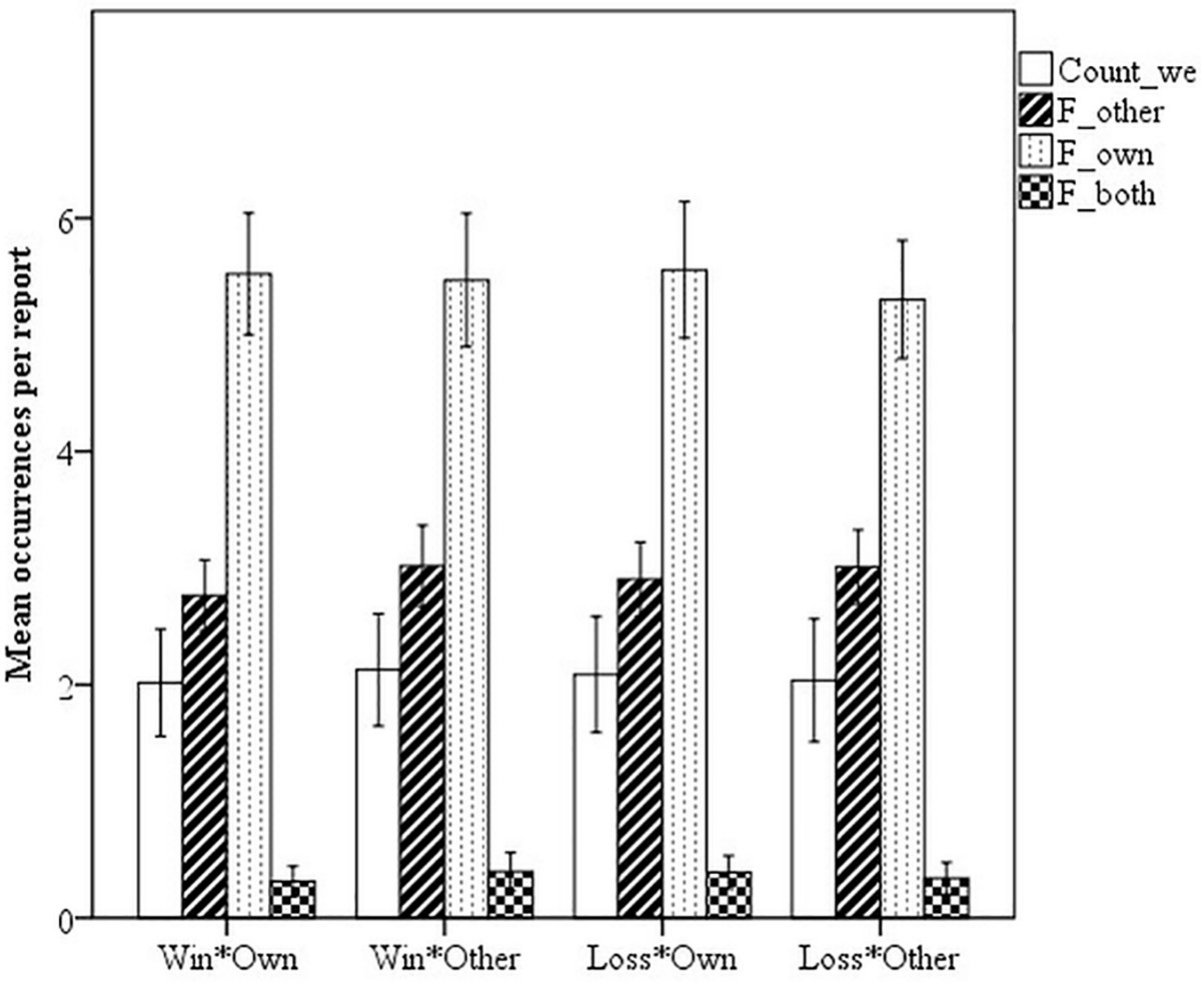
Average number of “we” and references to the own, other, or both teams per text by outcome and perspective; error bars are 95% CIs.

**Reference annotation.** With regard to the use of the pronoun *we* and author focus in the form of references to the own or the other team, or both teams, authors put focus equally on their own and the other team, as well as both teams, regardless of outcome or perspective (Fig 6).

### 2.6 Discussion

As shown above, participants’ affective states changed noticeably and congruently to the outcome of the game. Although, the outcome did not affect them in such a way that it caused strong negative or positive emotions, the effects were strong enough to affect the authors’ writing styles. Not only the use of positive and negative emotion words or words related to achievements varied as a function of affective state, which can be expected in a competitive game situation ending in success or failure, but also conceptual categories, including words linked to tentativeness and discrepancy, negations and even punctuation (exclamation marks).

Regarding the use of *we* and focus, participants showed no preference in either outcome or perspective. Since pronouns such as *we* have been shown to be good indicators of emotionality and social distance according to the literature, the lack of a difference in use could have different causes. The emotion induction might not have been strong enough to create a real in-group-out-group feeling or the instructions participants received to write the reports might have biased a specific text structure. Especially the latter can also be an explanation for the equal focus placed on both teams involved. We will investigate these matters further in Study 2.

The language in the reports and looking at the LIWC annotations, reveal an interesting aspect about linguistic focus in the reports about lost matches, see for example:

(15) OTHER_TEAM heeft deze keer gewoon geluk gehad. (10_R1_2T)

‘OTHER_TEAM was just lucky this time.’

(16) Het feit dat het andere team nu gewonnen heeft is puur geluk geweest. (24A_R1_1)

‘The fact that the other team won was pure luck.’

(17) Hoewel OTHER_TEAM met 10–5 hebben gewonnen, maakte OWN_TEAM hen het tot de laatste seconde moeilijk. (9A_R2_1OT)

‘Although OTHER_TEAM won with 10–5, OWN_TEAM put up a fight until the last second.’

In many of the match reports on lost matches, the authors summarize the outcome focusing on their opponent’s success, instead of explicitly mentioning the defeat of their own teams, see examples (15) to (17). Even authors assuming the losing teams’ perspectives employ this strategy. We will take a closer look at this distancing technique in Study 2.

## 3 Study 2

In Study 2, we set out to experimentally examine whether we did not find a difference in focus and the use of the pronoun *we* due to the formulation of the writing task in Study 1. Participants might have stayed too close to the text and content structure of the instructions (e.g., writing about the own and the other team was encouraged), which lead to the lack of differences. Instead of directed text production, participants in this study were asked to indicate which of a limited set of sentences best described the game they played.

### 3.1 Design

The design in the second experiment was a between-subjects design with one independent variable, Outcome (win, loss). Our dependent variables were affective state (questionnaire), basking/distancing (questionnaire and sentence choice of *we/team name*), and focus (own/other).

### 3.2 Participants

In total, 82 participants (54 women), with ages ranging from 17 to 33 years (*M* = 22.1), took part in the study. Participants were students and junior staff of Tilburg University who participated for course credit or candy. None of them had participated in the first study. All were fluent speakers of Dutch and partook again in groups of four, with one team consisting of two people. Consistent with the first study, we used same-sex groups. Two times, confederates were used to replace participants that did not show up. All but 4 participants had played foosball before and were familiar with the game. Neither the use of confederates, nor lack of experience with foosball had noticeable effects on the results.

### 3.3 Measures

The *Sport Emotion Questionnaire* and the *Basking and Distancing Questionnaire* from the first study remained the same in the follow-up study. To investigate preferred focus and basking/distancing, sentences from the foosball report corpus of Study 1 were selected.

#### 3.3.1 Basking/Distancing task

Participants could choose between two sentences that were identical except for the referring expression used to denote the subject, which was either “we” (inclusive, basking) or the team name (exclusive, distancing). In total, forty sentences were selected from the corpus. These sentences were divided into four bins of ten sentences each. Selection criteria were that the original sentence had to contain only one instance of “we” or the team name, and that the opponent’s team was not referred to by name or the third person plural “they”. To allow for more variety in the sentences, the other team could also be mentioned as “the opponent”, e.g.:

(18) We/TEAM_NAME gingen beschaamd het veld af en gaven de tegenstander nog een hand.

‘We/TEAM_NAME left the field ashamed but still shook hands with the opponent.’

#### 3.3.2 Focus task

Participants could choose between two sentences describing the same game event, but from either their own or the other teams perspective, e.g. “Team A won.” vs. “Team B lost.”. Again, forty sentences were collected from the corpus and divided into four bins of ten sentences each. In this case, the sentences could not contain pronouns in order to avoid ambiguity and priming for the basking/distancing sentences. Again, the original sentences from the corpus had to describe a game event from one team’s point of view, with the team name mentioned once. The matching sentences from the respective other team’s perspective were then written and approved by several Dutch native speakers, so:

(19) Uiteindelijk hebben TEAM_A de spannende wedstrijd gewonnen./Uiteindelijk hebben TEAM_B de spannende wedstrijd verloren.

‘In the end, TEAM_A won the exciting competition./In the end, TEAM_B lost the exciting competition.

(20) TEAM_A speelden beter en sneller./TEAM_B speelden slechter en trager.

‘TEAM_A played better and faster./TEAM_B played worse and more slowly.

#### 3.3.3 Fillers

Fillers consisted of twenty sentences and two categories that mirrored the structure of the critical sentences: alternative adjective sentences and full synonymous sentences. In the ten adjective fillers, participants could choose between two sentences that were identical except for one adjective, e.g.:

(21) Beide teams speelden fantastisch/uitmuntend.

‘Both teams played fantastically/excellently.’

In the ten full sentence fillers, participants could choose between two synonymous versions of a sentence. Fillers were not allowed to contain team names or pronouns referring to the teams in order to not interfere with the critical sentences. Overall, they described the game in general.

The four bins per critical sentence category resulted in four surveys. To randomize further, the order of the four basic surveys was reversed. In total, eight surveys with different sentence orders were used. Filler sentences stayed the same across conditions (win/loss) and survey versions.

### 3.4 Procedure

The procedure of the second experiment was similar to the first one, except for participants playing only one game of foosball. After the coin toss to determine team colors, each team could choose a team name from a selection of five names, each indicating a different team color. This approach was chosen to shorten the experiment and while still offering teams a personal choice. Participants then filled in the first part of the survey, which was the same as for Study 1. After the match, participants completed the sentence selection task. They were informed that they were to always choose between two different versions of automatically generated sentences about the match and, after making their choice, rate the chosen sentence on a five-point scale (bad–good). The rest of the survey and procedure remained the same as in the first study. Like for Study 1, the protocol was approved by the Tilburg School of Humanities and Digital Sciences Research Ethics and Data Management Committee, with written informed consent from all subjects.

### 3.5 Results

#### 3.5.1 Analysis strategy

For the analysis of self-reported emotions we used the same procedure, linear mixed effects modelling in R, as for Study 1 to account for the multilevel nature of the data. The questionnaire items were again collapsed into the five emotion categories. This time, the interaction of Time and Outcome was included as a predictor since we were interested in the difference between Win and Loss before and after the matches for both. Participants were included as random intercepts to account for repeated measures.

The five emotions (anger, anxiety, dejection, excitement, and happiness) were modelled separately as dependent variables, for example:

Dejection∼Outcome*Time+(1|Participant)

Again, we used parametric bootstrapping over 100 iterations to estimate confidence intervals, standard errors, and *p*-values on the models. For each model, we controlled whether gender, age, and familiarity with the other participants and the own teammate improved the model fit or influenced the Time/Outcome interaction.

As self-reported basking and distancing, for which the respective items were again collapsed, were only measured once, both were analyzed with an analysis of variance in R. Again, we controlled for possible effects of gender, age, and familiarity with the other participants and the own teammate.

The sentence selection task (Focus and Basking/Distancing) was analyzed with logit mixed modelling in R using the GLMER function of the lme4 package [50]. Following Winter [51], a bottom-up approach was used to build the models, starting with a basic model with game outcome as a fixed factor and participants as random intercepts, which was compared to a reduced model without the fixed factor. In a next step, we added random intercepts for items and random slopes for item by outcome to control for item variation. Adding participants as random slopes was again not possible due to the low number of observations for both self-reported measures and the linguistic task.

Finally, we checked whether gender, age, familiarity with the group, and the teammate improved the model and interacted with outcome.

For example, the final model for focus was as follows:

focus.model<‐glmer(Focus∼Outcome+(1|Participant)+(1+Outcome|Item),data=Data,family=binomial,control=glmerControl(optimizer="bobyqa"))

After every addition, the impact of the added factor was analyzed by comparing the full model to a reduced model without the new factor.

#### 3.5.2 Affective variables

**Sport emotion questionnaire.** Table 7 shows means and standard deviations for all five emotion categories in the win and loss conditions as well as the baseline values. Self-reported emotions differed significantly as a function of outcome. Compared to won matches (dejection: *M* = 1.10, *SD* = 0.37; anger: *M* = 1.03, *SD* = 0.08; excitement: *M* = 3.74, *SD* = 0.68; happiness: *M* = 3.90, *SD* = 0.78; anxiety: *M* = 1.15, *SD* = 0.43), reported dejection (*M* = 1.25, *SD* = 0.41, *b* = 0.20, *SE* = 0.06, BC 95% CI [0.08, 0.35]), anger (*M* = 1.26, *SD* = 0.56, *b* = 0.21, *SE* = 0.08, BC 95% CI [0.04, 0.38]), excitement (*M* = .3.54, *SD* = 0.82, *b* = -0.50, *SE* = 0.15, BC 95% CI [-0.81, -0.19]), and happiness (*M* = 3.78, *SD* = 0.79, *b* = -0.50, *SE* = 0.12, BC 95% CI [-0.75, -0.27]) were significantly different in the loss condition, indicating that winning again made participants happier and more exited, while they felt angrier and more dejected after losing (Table 8). These findings are consistent with Study 1.

**Table 7:** Descriptive statistics for emotions and basking/distancing in baseline, loss, and win.

	BASELINE	WIN	LOSS
	M (SD)	M (SD)	M (SD)
Anger	1.12 (0.28)	1.03 (0.08)	1.26 (0.56)
Anxiety	1.39 (0.49)	1.15 (0.43)	1.27 (0.43)
Dejection	1.20 (0.40)	1.10 (0.37)	1.25 (0.41)
Excitement	3.15 (0.79)	3.74 (0.68)	3.54 (0.82)
Happiness	3.57 (0.76)	3.90 (0.78)	3.78 (0.79)
Basking	—	4.38 (0.42)	3.58 (0.69)
Distancing	—	1.44 (0.45)	1.76 (0.84)

**Table 8:** Analyses for all five emotions (anxiety, dejection, excitement, happiness, anger).

	*B*	*SE b*	*95% CI*
**Anxiety**			
Intercept	1.37	0.07	1.21, 1.52
Outcome Loss	0.04	0.11	-0.18, 0.27
Time 2	-0.21	0.05	-0.32, - 0.09
Outcome Loss–Time 2	0.06	0.08	-0.10, 0.24
**Dejection**			
Intercept	1.22	0.06	1.10, 1.34
Outcome Loss	-0.05	0.08	-0.21, 0.11
Time 2	-0.12	0.04	-0.22, -0.04
**Outcome Loss–Time 2**	**0.20**	**0.06**	**0.08, 0.35**
**Excitement**			
Intercept	3.00	0.11	2.80, 3.24
Outcome Loss	0.29	0.17	-0.08, 0.60
Time 2	0.74	0.10	0.53, 0.93
**Outcome Loss–Time 2**	**-0.50**	**0.15**	**-0.81, -0.19**
**Happiness**			
Intercept	3.37	0.10	3.16, 3.58
Outcome Loss	0.37	0.16	0.06, 0.72
Time 2	0.52	0.08	0.35, 0.69
**Outcome Loss–Time 2**	**-0.50**	**0.12**	**-0.75, -0.27**
**Anger**			
Intercept	1.11	0.05	1.00, 1.22
Outcome Loss	0.01	0.07	-0.14, 0.15
Time 2	-0.07	0.05	-0.18, 0.02
**Outcome Loss–Time 2**	**0.21**	**0.08**	**0.04, 0.38**

Estimated coefficients B, standard errors SE b, and 95% confidence intervals for the full mixed models; participants as random intercepts; interaction of game outcome and time as the predictor; win as the reference category. Significant interactions are represented in bold.

Similar to the first study, there was no interaction effect of time and outcome on the level of anxiety reported: anxiety only reduced over time and was influenced by participants’ gender. Including gender also improved the models for anger and dejection, but it did not impact the interaction of outcome and time, and, hence, did not change the results. Male participants were simply angrier after lost matches, and experienced more dejection and anxiety in general compared to female participants. Group familiarity, age, and the familiarity with one’s teammate did not improve any of the models.

**Basking and distancing questionnaire.** As before, winners and losers differed (Table 7) both in self-reported basking (*F*(1, 80) = 40.02, *p* < .001) and distancing (*F*(1, 78) = 4.75, *p* = .022). Again, participants generally bask more than they distance themselves, but basking behavior additionally increases after a won match and drops after a loss, while the opposite is true for distancing. Additionally, adding familiarity with the teammate or the whole group did not have an effect on basking and distancing.

**Fig 7:**
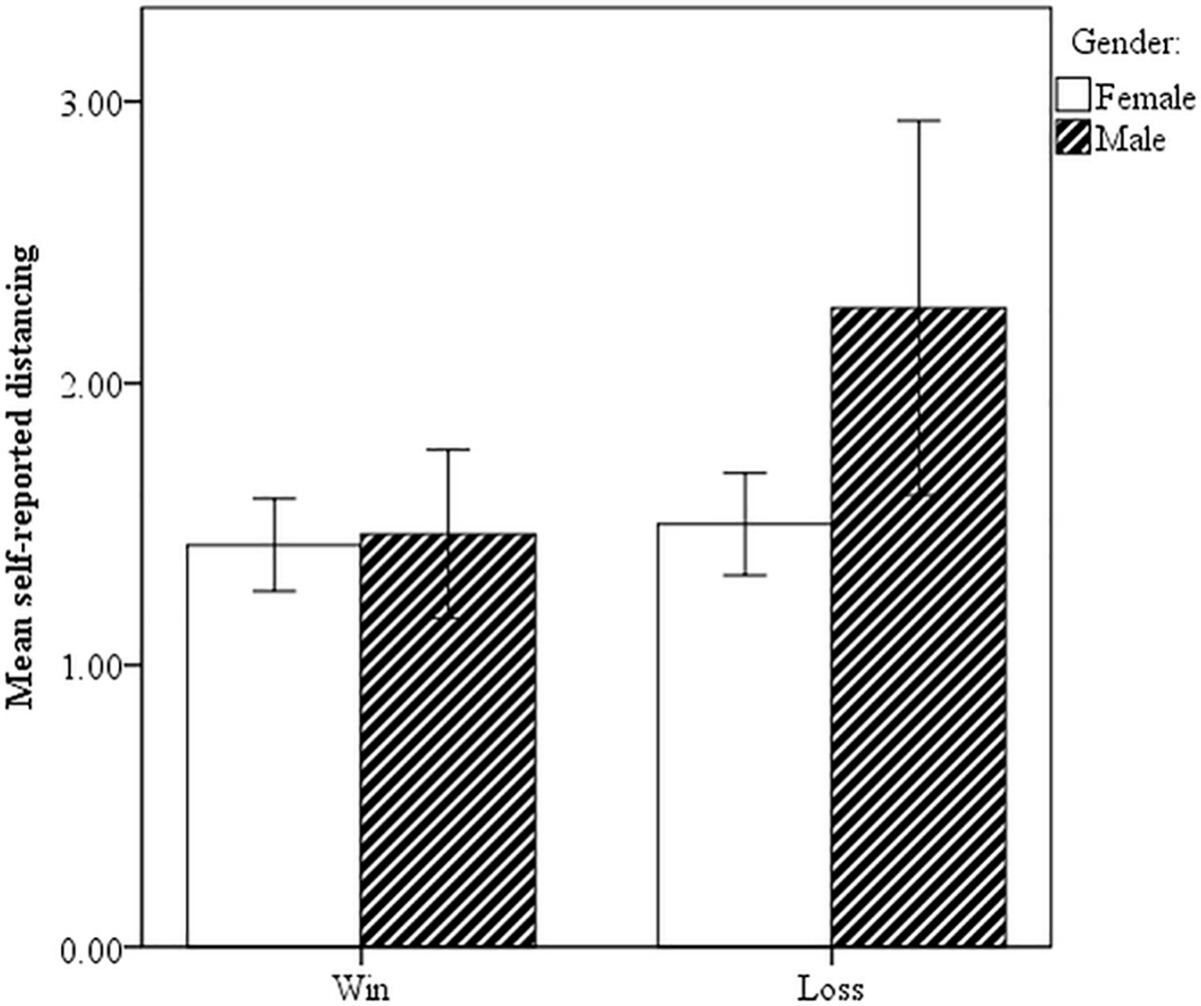
Means of distancing by gender; error bars are 95% CIs.

However, gender had a significant main effect (*F*(1, 78) = 7.63, *p* = .007) and interacted with outcome (*F*(1, 78) = 6.24, *p* = .015) on distancing. Male participants distanced themselves much more from a lost match than female participants (see Fig 7).

#### 3.5.3 Sentence selection task

Basic descriptive statistics for focus and basking/distancing can be found in Table 9.

**Table 9:** Means and Standard deviations for the proportion of focus (0 = Own, 1 = Other) and basking/ distancing (0 = We, 1 = team name).

	Win (SD)	Loss (SD)
*we* vs. team name	0.55(.49)	0.53(0.50)
Focus	0.18(.38)	0.62 (0.48)

**Fig 8:**
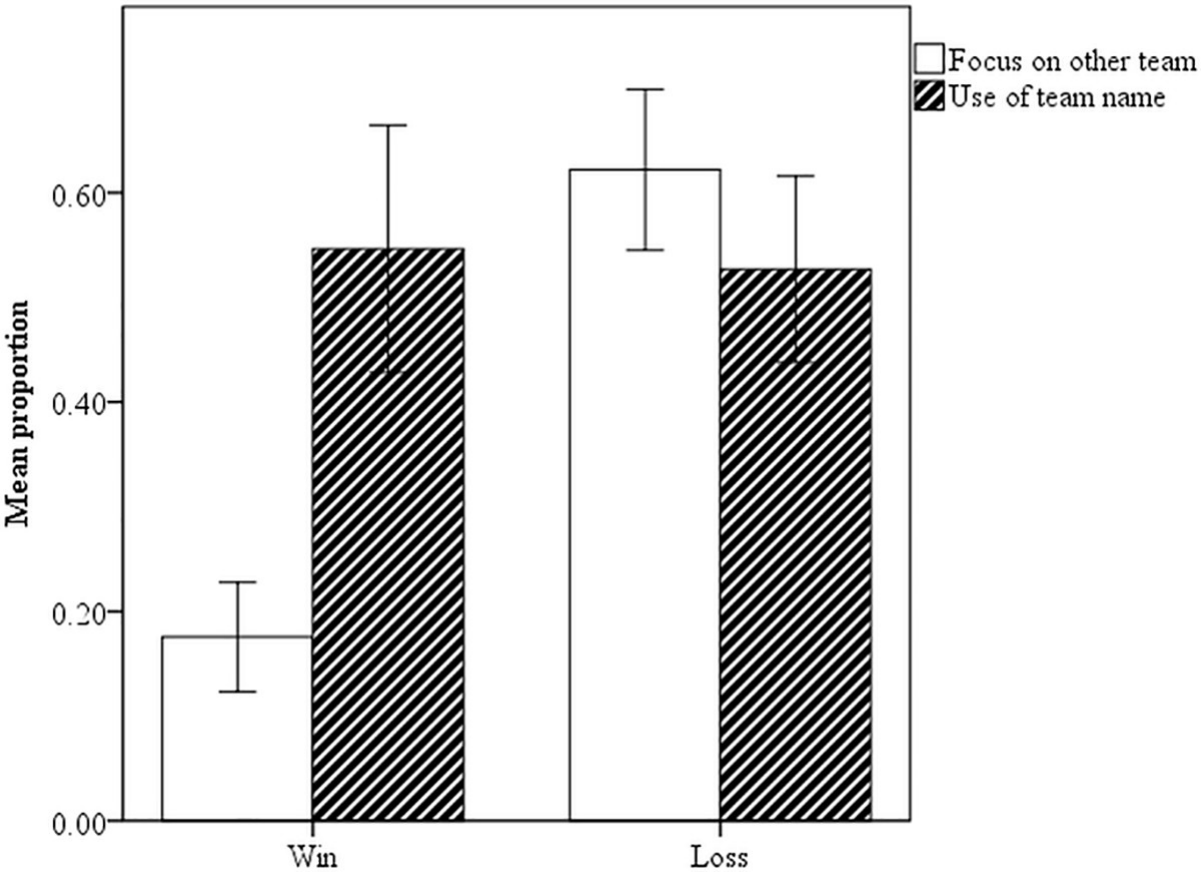
Means for the proportion of focus (0 = own, 1 = other) and *we* (0 = *we*, 1 = team name) in Win and Loss; error bars are 95% CIs.

**Basking/distancing.** There was no significant difference between won matches (*M* = 0.55, *SD* = 0.49) and lost matches (*M* = 0.53, *SD* = 0.50, *b* = -0.20, *SE* = 0.48, BC 95% CI [-1.19, 0.71]) so there is no evidence that game outcome changed participants’ choices for pronoun or team name (Table 10): participants equally often chose sentences with team name and the pronoun *we* to describe the match (Fig 8). For the sake of completeness, additional factors were included to see whether this improved the model, i.e., the familiarity with the group, familiarity with the own teammate, age, and gender. Although both, the intermediate models as well as the complete model converged, overall model fit worsened for the complete model. In addition, excluding groups with confederates did not change the above mentioned results. Hence, the fact that occasionally a confederate had to be used did not influence the results.

**Table 10:** Analyses for focus and basking (we vs. team name).

	*B*	*SE b*	*95% CI*
*we* vs. team name			
Intercept	0.36	0.33	-0.28, 1.03
Outcome Loss	-0.20	0.48	-1.19, 0.71
**Focus**			
Intercept	-2.28	0.41	-3.11, -1.50
Outcome Loss	2.91	0.48	1.99, 3.92

Estimated coefficients B, standard errors SE b, and 95% confidence intervals for the full mixed models; participants as random intercepts; item by outcome as random slopes; outcome as the predictor. A comparison with reduced models proved that the inclusion of items as random intercepts did not improve the models.

**Focus.** In contrast to basking, outcome predicted focus (Table 10). Winners (*M* = 0.18, *SD* = 0.38) chose sentences about the losing team’s performance significantly less than losers (M = 0.62, SD = 0.48, b = 2.91, SE = 0.48, BC 95% CI [1.99, 3.92]) chose sentences about the winning team (Fig 8).

Again, familiarity with the other participants, familiarity with the own teammate, age, and gender were added as factors. However, neither of factors yielded a significant model improvement.

### 3.6 Discussion

Similar to Study 1, participants’ emotions changed congruent with game outcome: after a lost match, participants felt angrier and more dejected, and after a won match, they felt happier and more excited. They also expressed more self-reported distancing after a loss than after a win, and more basking behavior after a win. However, also like in Study 1, we did not observe a preference for the pronoun *we* after a success or a preference for team names after a lost match. Participants used both kinds of references approximately equally often. Contrastingly, focus depended strongly on game outcome. Participants in losing teams preferred sentences describing their opponents’ performances over sentences describing their own teams’ performances negatively in more than 60% of the cases. Winners only chose to talk about their opponent in 20% of the cases. This coincides with self-reported basking/distancing and the self-reported emotions experienced after the game: participants did not only report feeling more distant from their team after a lost match, but they also distanced themselves in terms of focus. It can argued that this is serves as a kind of self-preservation mechanism. So, instead of “bashing” their own team and performance in the game, participants prefer highlighting the superiority of the other team, which serves as an explanation for the outcome and as a distractor from themselves.

## 4 General discussion

The goal of our studies was to create a paradigm with a natural setting in the lab for competitive play. In an earlier study, we investigated the role of positive and negative affect (in the form of winning and losing) on language in a multilingual text corpus of soccer reports that were harvested from the involved clubs’ websites [52]. We indeed showed relationships between game outcome and affective language use. However, it was difficult to attribute authorship in online match reports and it was equally difficult to gauge the affiliation of the author, who was perhaps a professional writer writing for a club’s website, but who was otherwise detached from the club [52]. To avoid these issues, we decided to recreate a comparable setup in the lab with foosball, similar to the children’s soccer study of Baker-Ward and Eaton [27]. We wanted to investigate whether it is possible to change the affective state of participants with competitive play and that these changes, just like the different perspectives that people assume, affect other cognitive processes, in our case language production. Since the competition took place in a group, we additionally expected phenomena such as basking in successes [19] and distancing from failures [38] to influence language production and group dynamics.

Overall, the findings confirm the relation between affect, language, and perspective. Study 1 explored the influence of competitive foosball on participants’ affective states, group cohesion, and the way participants reported on the game itself. We showed that success and failure in the foosball matches succeeded as an affect induction method to induce mild negative and positive affect in our participants. Winning participants reported feeling happier and more excited, losing participants indicated more dejection and anger. At the same time, they distanced themselves more from their groups after a lost match, while basking more after winning. Basking and distancing also correlated with the respective emotions. Further, these changes were also traceable in the match reports that participants wrote about the matches afterwards.

With a word count [11], we showed changes in different language categories that are relate to affect. While reports generally did not differ in text length or writing time regardless of game outcome or perspective taken, more subtle linguistic categories varied. On the one hand, participants not only seem to adapt straightforward linguistic aspects such as positive and negative emotion words, but they also changed their writing in terms of tentativeness, discrepancy, or punctuation (exclamation marks). Whether these linguistic changes happen consciously or subconsciously stands to question.

Consciously or not, authors adapted their reports and writing styles intuitively also when they assumed their opponent’s perspective, without being cued to pay attention to specific linguistic categories. Yet, there seem to exist subtle language differences in describing a success or a failure that authors were not aware of, since they did not adapt their writing in terms of negations or tentative language.

At the same time, we noticed some limitations of LIWC itself. Since the tool does not take textual context into account, some words were overlooked or misclassified in our analysis [53]. The impact of this issue should be investigated more closely in the future.

Contrary to our expectations, we found no difference in the number of references to the authors’ own teams and the other teams. Author focus was roughly equally distributed over both teams for both outcomes. Based on literature about basking and distancing behavior and psychological distancing, a stronger focus on one’s own team after a won match and more distant linguistic variants after a lost match would have appeared logical.

In addition, we believe that the mildness of the self-reported emotions could be an order effect, related to the fact that participants wrote the reports before indicating their emotions. Similar to work on using writing as a form of therapy in the treatment of depression [44, 45, 54], writing the match reports might have also dampened the experience of emotions after the task and, hence, also the self-report. We think that this could be an interesting starting point for future work.

Similarly, no difference in reference type to the own team (*we* vs. team name) was found for either outcome or perspective, despite the fact that literature on basking and distancing also suggests a more frequent use of the 1^st^ person plural as a means of self-inclusion and self-serving in successes [19]. We suspected that the phrasing of the writing task could have primed a specific text and reference structure, which we investigated further in the second study.

Study 2 confirmed the game as a natural affect induction method and tested the effect on participants’ affective state on a more controlled, but still closely related linguistic task. Participants were again happier and more excited after a won match, while they reported more dejection and anger after a lost match. Additionally, we also found the same pattern for basking and distancing behavior.

These changes in affect and the basking/distancing behavior seemed to further influence the linguistic task. Based on the corpus derived from Study 1, participants chose sentence focus (own team/other team) and reference type (*we*/team name). Still, we found no preference for the pronoun *we* after won matches or for team name, the more distant option, after a defeat. Reference choice appeared to be a personal preference. Unaffected by game outcome, some participants used only *we*, while others preferred using team name continuously, regardless of game outcome or familiarity with the group. Again others chose *we* and team name equally often, as if to alternate stylistically although they only chose between individual sentences. Naturally, people prefer variation in references (see, e.g., [55]), which might be an explanation for the individual variation in reference choice. Furthermore, in contrast to (professional) athletes, our studies did not investigate participants with a strong affiliation to the game they played, the team they played in or with a strong external motivation to win. Moreover, it is likely that the personality of individuals plays a role in the way they handle and talk about failures and successes: a sore loser probably reports differently about a lost match than someone who is naturally not as troubled by failures.

Further, we did find an effect of game outcome on preferred focus. In the sentence selection task, winners selected the sentences about their own team in more than 80% of the cases, while losers only did so for less than 40% of sentence pairs. Naturally, it would be reasonable to assume that each participant chooses descriptions about their own team’s performance if given the choice. Yet, in some cases, winners chose to put the focus on the losing team, and, in the majority of cases, losers preferred to also focus on the winning team. Since the sentences that were used for the task were adapted to game outcome, winning team sentences described a positive game outcome and can thereby be considered positive themselves, while losing team sentences described a negative game outcome and were rather negative. Given that (psychological) distance is commonly employed to regulate emotions [42, 56, 57], it is reasonable to assume that losing participants would prefer to not report about their own performance in a negative way and resort to “praising” their opponents. By talking about their own team, they would have signaled involvement in the outcome, which they likely avoided. This additionally serves as a way to create distance from the event and to distract from one’s own performance. On the other side, winners might focus on the losing team in order to bask in their own success by mocking their opponent.

In sum, we managed to demonstrate a new naturalistic affect induction procedure in a social setting with a competitive game and presented tasks that are closely connected to the induction method. We also showed how subtle changes in the affective state influence language production–in our case focus and specific word categories. In addition, we illustrated how mechanisms such as self-serving and self-preservation can influence these levels of language production in terms of basking and distancing language.

## Supporting information

S1 Table Correlations of LIWC categories and self-reported basking/distancing. (PDF)

S2 Table Correlations of LIWC categories (pronoun, we, negations, positive emotions, negative emotions, anxiety, anger, sadness, discrepancy, inhibition, tentativeness, certainty, achievement, exclamation marks) and self-reported emotions (anxiety, dejection, excitement, anger, happiness). (PDF)

S1 File README file for S1 Table. (TXT)

S2 File README file for S2 Table. (TXT)

S1 Dataset Study 1 data for emotions and basking/distancing. (XLSX)

S2 Dataset Study 1 data for LIWC. (XLSX)

S3 Dataset Study 2 data. (XLSX)
