# Supplementary material for: Language and Emotion – A Foosball Study: The Influence of Affective State on Language Production in a Competitive Setting
Source: PLoS One. 2019 May 24;14(5):e0217419. doi: 10.1371/journal.pone.0217419 (PMC6534325; doi:10.1371/journal.pone.0217419)
Supplement: S1 Table — (PDF) [file pone.0217419.s001.pdf]

S1 Table. Correlations of LIWC Categories and Self-Reported Basking/Distancing.

		Basking	Distancing	Pronoun	We	Negate	Posemo	Negemo	Anx	Anger	Sad	Discrep	Inhib	Tentat	Certain	Achieve	Exclam
Basking	Pearson Correlation	1	-.521**	.019	.007	-.063	-.027	-.001	.007	.043	-.083	-.093*	.113*	-.049	-.050	-.058	-.008
	Sig. (2-tailed)		.000	.682	.880	.181	.572	.981	.880	.361	.078	.050	.017	.295	.288	.220	.859
	N	450	450	450	450	450	450	450	450	450	450	450	450	450	450	450	450
Distancing	Pearson Correlation	-.521**	1	-.061	-.020	.104*	.042	.035	-.004	.065	.041	.026	.007	-.008	.099*	-.009	.065
	Sig. (2-tailed)	.000		.200	.675	.027	.373	.457	.931	.170	.383	.581	.878	.871	.036	.845	.167
	N	450	450	450	450	450	450	450	450	450	450	450	450	450	450	450	450
Pronoun	Pearson Correlation	.019	-.061	1	.722**	.020	.195**	.062	-.127**	.026	.056	.102*	-.074	-.073	.125**	.142**	.205**
	Sig. (2-tailed)	.682	.200		.000	.668	.000	.188	.007	.588	.235	.031	.119	.122	.008	.003	.000
	N	450	450	450	450	450	450	450	450	450	450	450	450	450	450	450	450
We	Pearson Correlation	.007	-.020	.722**	1	.036	.230**	.132**	-.091	.095*	.089	.067	-.016	-.058	.037	.142**	.162**
	Sig. (2-tailed)	.880	.675	.000		.445	.000	.005	.053	.045	.059	.159	.728	.216	.437	.003	.001
	N	450	450	450	450	450	450	450	450	450	450	450	450	450	450	450	450
Negate	Pearson Correlation	-.063	.104*	.020	.036	1	.019	.130**	-.065	.055	.150**	.103*	.118*	.032	.135**	-.047	.016
	Sig. (2-tailed)	.181	.027	.668	.445		.684	.006	.169	.245	.001	.029	.012	.498	.004	.315	.730
	N	450	450	450	450	450	450	450	450	450	450	450	450	450	450	450	450
Posemo	Pearson Correlation	-.027	.042	.195**	.230**	.019	1	-.003	-.111*	.063	-.059	.102*	.057	.106*	.116*	.431**	.097*
	Sig. (2-tailed)	.572	.373	.000	.000	.684		.947	.018	.183	.215	.030	.225	.025	.014	.000	.039
	N	450	450	450	450	450	450	450	450	450	450	450	450	450	450	450	450
Negemo	Pearson Correlation	-.001	.035	.062	.132**	.130**	-.003	1	.196**	.618**	.509**	.110*	.266**	.033	-.053	.101*	-.066
	Sig. (2-tailed)	.981	.457	.188	.005	.006	.947		.000	.000	.000	.020	.000	.479	.264	.033	.163
	N	450	450	450	450	450	450	450	450	450	450	450	450	450	450	450	450
Anx	Pearson Correlation	.007	-.004	-.127**	-.091	-.065	-.111*	.196**	1	.048	.013	-.033	-.090	.008	-.034	-.073	-.056
	Sig. (2-tailed)	.880	.931	.007	.053	.169	.018	.000		.313	.785	.488	.057	.865	.470	.123	.239
	N	450	450	450	450	450	450	450	450	450	450	450	450	450	450	450	450
Anger	Pearson Correlation	.043	.065	.026	.095*	.055	.063	.618**	.048	1	-.037	-.010	.547**	-.011	-.068	-.030	.032
	Sig. (2-tailed)	.361	.170	.588	.045	.245	.183	.000	.313		.434	.835	.000	.817	.152	.522	.499
	N	450	450	450	450	450	450	450	450	450	450	450	450	450	450	450	450
Sad	Pearson Correlation	-.083	.041	.056	.089	.150**	-.059	.509**	.013	-.037	1	.141**	-.011	.059	.015	.283**	-.092
	Sig. (2-tailed)	.078	.383	.235	.059	.001	.215	.000	.785	.434		.003	.818	.209	.755	.000	.052
	N	450	450	450	450	450	450	450	450	450	450	450	450	450	450	450	450
Discrep	Pearson Correlation	-.093*	.026	.102*	.067	.103*	.102*	.110*	-.033	-.010	.141**	1	-.022	.023	.122**	.048	.013
	Sig. (2-tailed)	.050	.581	.031	.159	.029	.030	.020	.488	.835	.003		.647	.625	.009	.314	.789
	N	450	450	450	450	450	450	450	450	450	450	450	450	450	450	450	450
Inhib	Pearson Correlation	.113*	.007	-.074	-.016	.118*	.057	.266**	-.090	.547**	-.011	-.022	1	-.037	-.077	.039	.027
	Sig. (2-tailed)	.017	.878	.119	.728	.012	.225	.000	.057	.000	.818	.647		.437	.101	.412	.564

	N	450	450	450	450	450	450	450	450	450	450	450	450	450	450	450	450
Tentat	Pearson Correlation	-.049	-.008	-.073	-.058	.032	.106*	.033	.008	-.011	.059	.023	-.037	1	-.030	-.032	-.119*
	Sig. (2-tailed)	.295	.871	.122	.216	.498	.025	.479	.865	.817	.209	.625	.437		.531	.498	.012
	N	450	450	450	450	450	450	450	450	450	450	450	450	450	450	450	450
Certain	Pearson Correlation	-.050	.099*	.125**	.037	.135**	.116*	-.053	-.034	-.068	.015	.122**	-.077	-.030	1	.049	-.004
	Sig. (2-tailed)	.288	.036	.008	.437	.004	.014	.264	.470	.152	.755	.009	.101	.531		.299	.930
	N	450	450	450	450	450	450	450	450	450	450	450	450	450	450	450	450
Achieve	Pearson Correlation	-.058	-.009	.142**	.142**	-.047	.431**	.101*	-.073	-.030	.283**	.048	.039	-.032	.049	1	.029
	Sig. (2-tailed)	.220	.845	.003	.003	.315	.000	.033	.123	.522	.000	.314	.412	.498	.299		.541
	N	450	450	450	450	450	450	450	450	450	450	450	450	450	450	450	450
Exclam	Pearson Correlation	-.008	.065	.205**	.162**	.016	.097*	-.066	-.056	.032	-.092	.013	.027	-.119*	-.004	.029	1
	Sig. (2-tailed)	.859	.167	.000	.001	.730	.039	.163	.239	.499	.052	.789	.564	.012	.930	.541	
	N	450	450	450	450	450	450	450	450	450	450	450	450	450	450	450	450

**. Correlation is significant at the 0.01 level (2-tailed).

*. Correlation is significant at the 0.05 level (2-tailed).
