# Supplementary material for: Language and Emotion – A Foosball Study: The Influence of Affective State on Language Production in a Competitive Setting
Source: PLoS One. 2019 May 24;14(5):e0217419. doi: 10.1371/journal.pone.0217419 (PMC6534325; doi:10.1371/journal.pone.0217419)
Supplement: S2 Table — (PDF) [file pone.0217419.s002.pdf]

S2 Table. Correlations of LIWC Categories (Pronoun, We, Negations, Positive Emotions, Negative Emotions, Anxiety, Anger, Sadness, Discrepancy, Inhibition, Tentativeness, Certainty, Achievement, Exclamation Marks) and Self-Reported Emotions (Anxiety, Dejection, Excitement, Anger, Happiness).

		Anxiety	Dejection	Excitement	Anger	Happiness	Pronoun	We	Negate	Posemo	Negemo	Anx	Anger	Sad	Discrep	Inhib	Tentat	Certain	Achieve	Exclam
Anxiety	Pearson Correlation	1	.438**	-.008	.394**	-.043	-.076	.009	-.033	-.082	-.153**	.040	-.151**	-.020	-.049	-.123**	.014	.004	-.062	-.037
	Sig. (2-tailed)		.000	.872	.000	.367	.105	.850	.488	.081	.001	.395	.001	.672	.302	.009	.768	.929	.187	.431
	N	450	450	450	450	450	450	450	450	450	450	450	450	450	450	450	450	450	450	450
Dejection	Pearson Correlation	.438**	1	-.270**	.671**	-.359**	-.049	-.035	.031	-.104*	-.037	.022	-.109*	.131**	.075	-.081	.033	.122**	-.057	-.068
	Sig. (2-tailed)	.000		.000	.000	.000	.298	.459	.509	.028	.435	.644	.020	.005	.114	.084	.486	.010	.228	.149
	N	450	450	450	450	450	450	450	450	450	450	450	450	450	450	450	450	450	450	450
Excitement	Pearson Correlation	-.008	-.270**	1	-.226**	.806**	.071	.031	-.042	-.009	-.005	.007	.000	-.139**	-.072	.011	-.058	-.074	-.119*	.002
	Sig. (2-tailed)	.872	.000		.000	.000	.132	.518	.370	.849	.915	.875	.994	.003	.125	.818	.218	.115	.011	.967
	N	450	450	450	450	450	450	450	450	450	450	450	450	450	450	450	450	450	450	450
Anger	Pearson Correlation	.394**	.671**	-.226**	1	-.296**	-.047	-.046	-.016	-.092	.012	-.013	-.067	.113*	.034	-.105*	.016	.132**	.045	-.006
	Sig. (2-tailed)	.000	.000	.000		.000	.319	.332	.733	.050	.792	.784	.153	.016	.468	.025	.742	.005	.345	.895
	N	450	450	450	450	450	450	450	450	450	450	450	450	450	450	450	450	450	450	450
Happiness	Pearson Correlation	-.043	-.359**	.806**	-.296**	1	.104*	.067	.020	-.050	-.012	-.001	.000	-.105*	-.065	.053	-.042	-.064	-.104*	.059
	Sig. (2-tailed)	.367	.000	.000	.000		.028	.157	.668	.294	.806	.988	.993	.026	.167	.266	.373	.178	.027	.214
	N	450	450	450	450	450	450	450	450	450	450	450	450	450	450	450	450	450	450	450
Pronoun	Pearson Correlation	-.076	-.049	.071	-.047	.104*	1	.722**	.020	.195**	.062	-.127**	.026	.056	.102*	-.074	-.073	.125**	.142**	.205**
	Sig. (2-tailed)	.105	.298	.132	.319	.028		.000	.668	.000	.188	.007	.588	.235	.031	.119	.122	.008	.003	.000
	N	450	450	450	450	450	450	450	450	450	450	450	450	450	450	450	450	450	450	450
We	Pearson Correlation	.009	-.035	.031	-.046	.067	.722**	1	.036	.230**	.132**	-.091	.095*	.089	.067	-.016	-.058	.037	.142**	.162**
	Sig. (2-tailed)	.850	.459	.518	.332	.157	.000		.445	.000	.005	.053	.045	.059	.159	.728	.216	.437	.003	.001
	N	450	450	450	450	450	450	450	450	450	450	450	450	450	450	450	450	450	450	450
Negate	Pearson Correlation	-.033	.031	-.042	-.016	.020	.020	.036	1	.019	.130**	-.065	.055	.150**	.103*	.118*	.032	.135**	-.047	.016
	Sig. (2-tailed)	.488	.509	.370	.733	.668	.668	.445		.684	.006	.169	.245	.001	.029	.012	.498	.004	.315	.730
	N	450	450	450	450	450	450	450	450	450	450	450	450	450	450	450	450	450	450	450
Posemo	Pearson Correlation	-.082	-.104*	-.009	-.092	-.050	.195**	.230**	.019	1	-.003	-.111*	.063	-.059	.102*	.057	.106*	.116*	.431**	.097*
	Sig. (2-tailed)	.081	.028	.849	.050	.294	.000	.000	.684		.947	.018	.183	.215	.030	.225	.025	.014	.000	.039
	N	450	450	450	450	450	450	450	450	450	450	450	450	450	450	450	450	450	450	450
Negemo	Pearson Correlation	-.153**	-.037	-.005	.012	-.012	.062	.132**	.130**	-.003	1	.196**	.618**	.509**	.110*	.266**	.033	-.053	.101*	-.066
	Sig. (2-tailed)	.001	.435	.915	.792	.806	.188	.005	.006	.947		.000	.000	.000	.020	.000	.479	.264	.033	.163
	N	450	450	450	450	450	450	450	450	450	450	450	450	450	450	450	450	450	450	450
Anx	Pearson Correlation	.040	.022	.007	-.013	-.001	-.127**	-.091	-.065	-.111*	.196**	1	.048	.013	-.033	-.090	.008	-.034	-.073	-.056
	Sig. (2-tailed)	.395	.644	.875	.784	.988	.007	.053	.169	.018	.000		.313	.785	.488	.057	.865	.470	.123	.239
	N	450	450	450	450	450	450	450	450	450	450	450	450	450	450	450	450	450	450	450

Anger	Pearson Correlation	-.151**	-.109*	.000	-.067	.000	.026	.095*	.055	.063	.618**	.048	1	-.037	-.010	.547**	-.011	-.068	-.030	.032
	Sig. (2-tailed)	.001	.020	.994	.153	.993	.588	.045	.245	.183	.000	.313		.434	.835	.000	.817	.152	.522	.499
	N	450	450	450	450	450	450	450	450	450	450	450	450	450	450	450	450	450	450	450
Sad	Pearson Correlation	-.020	.131**	-.139**	.113*	-.105*	.056	.089	.150**	-.059	.509**	.013	-.037	1	.141**	-.011	.059	.015	.283**	-.092
	Sig. (2-tailed)	.672	.005	.003	.016	.026	.235	.059	.001	.215	.000	.785	.434		.003	.818	.209	.755	.000	.052
	N	450	450	450	450	450	450	450	450	450	450	450	450	450	450	450	450	450	450	450
Discrep	Pearson Correlation	-.049	.075	-.072	.034	-.065	.102*	.067	.103*	.102*	.110*	-.033	-.010	.141**	1	-.022	.023	.122**	.048	.013
	Sig. (2-tailed)	.302	.114	.125	.468	.167	.031	.159	.029	.030	.020	.488	.835	.003		.647	.625	.009	.314	.789
	N	450	450	450	450	450	450	450	450	450	450	450	450	450	450	450	450	450	450	450
Inhib	Pearson Correlation	-.123**	-.081	.011	-.105*	.053	-.074	-.016	.118*	.057	.266**	-.090	.547**	-.011	-.022	1	-.037	-.077	.039	.027
	Sig. (2-tailed)	.009	.084	.818	.025	.266	.119	.728	.012	.225	.000	.057	.000	.818	.647		.437	.101	.412	.564
	N	450	450	450	450	450	450	450	450	450	450	450	450	450	450	450	450	450	450	450
Tentat	Pearson Correlation	.014	.033	-.058	.016	-.042	-.073	-.058	.032	.106*	.033	.008	-.011	.059	.023	-.037	1	-.030	-.032	-.119*
	Sig. (2-tailed)	.768	.486	.218	.742	.373	.122	.216	.498	.025	.479	.865	.817	.209	.625	.437		.531	.498	.012
	N	450	450	450	450	450	450	450	450	450	450	450	450	450	450	450	450	450	450	450
Certain	Pearson Correlation	.004	.122**	-.074	.132**	-.064	.125**	.037	.135**	.116*	-.053	-.034	-.068	.015	.122**	-.077	-.030	1	.049	-.004
	Sig. (2-tailed)	.929	.010	.115	.005	.178	.008	.437	.004	.014	.264	.470	.152	.755	.009	.101	.531		.299	.930
	N	450	450	450	450	450	450	450	450	450	450	450	450	450	450	450	450	450	450	450
Achieve	Pearson Correlation	-.062	-.057	-.119*	.045	-.104*	.142**	.142**	-.047	.431**	.101*	-.073	-.030	.283**	.048	.039	-.032	.049	1	.029
	Sig. (2-tailed)	.187	.228	.011	.345	.027	.003	.003	.315	.000	.033	.123	.522	.000	.314	.412	.498	.299		.541
	N	450	450	450	450	450	450	450	450	450	450	450	450	450	450	450	450	450	450	450
Exclam	Pearson Correlation	-.037	-.068	.002	-.006	.059	.205**	.162**	.016	.097*	-.066	-.056	.032	-.092	.013	.027	-.119*	-.004	.029	1
	Sig. (2-tailed)	.431	.149	.967	.895	.214	.000	.001	.730	.039	.163	.239	.499	.052	.789	.564	.012	.930	.541	
	N	450	450	450	450	450	450	450	450	450	450	450	450	450	450	450	450	450	450	450

**. Correlation is significant at the 0.01 level (2-tailed).

*. Correlation is significant at the 0.05 level (2-tailed).
